# Differential Expression of Serum Extracellular Vesicle miRNAs in Multiple Sclerosis: Disease-Stage Specificity and Relevance to Pathophysiology

**DOI:** 10.3390/ijms23031664

**Published:** 2022-01-31

**Authors:** Nagiua Cuomo-Haymour, Giorgio Bergamini, Giancarlo Russo, Luka Kulic, Irene Knuesel, Roland Martin, André Huss, Hayrettin Tumani, Markus Otto, Christopher R. Pryce

**Affiliations:** 1 Preclinical Laboratory for Translational Research into Affective Disorders, Department of Psychiatry, Psychotherapy and Psychosomatics, Psychiatric Hospital, University of Zurich, 8008 Zurich, Switzerland; nagiua.haymour@bli.uzh.ch (N.C.-H.); bergamini.giorgio@gmail.com (G.B.); 2 Neuroscience Center Zurich, 8057 Zurich, Switzerland; 3 Functional Genomics Centre Zurich, University of Zurich and Swiss Federal Institute of Technology Zurich, 8057 Zurich, Switzerland; giancarlo1russo@gmail.com; 4 Roche Innovation Center Basel, Neuroimmunology Division, Roche Pharma Research and Early Development, 4070 Basel, Switzerland; luka.kulic@roche.com (L.K.); knuesel@me.com (I.K.); 5 Neuroimmunology and MS Research, Neurology Clinic, University Hospital Zurich, 8006 Zurich, Switzerland; roland.martin@usz.ch; 6 Department of Neurology, University Hospital Ulm, 89081 Ulm, Germany; andre.huss@uni-ulm.de (A.H.); hayrettin.tumani@uni-ulm.de (H.T.); 7 Department of Neurology, University Hospital Halle, Martin Luther University Halle-Wittenberg, 06120 Halle (Saale), Germany; markus.otto@uk-halle.de

**Keywords:** clinically isolated syndrome, relapsing–remitting multiple sclerosis, inflammation, extracellular vesicles, microRNA, biomarker, pathophysiology

## Abstract

Multiple sclerosis (MS) is a chronic inflammatory autoimmune disease of the central nervous system (CNS). Its first clinical presentation (clinically isolated syndrome, CIS) is often followed by the development of relapsing–remitting MS (RRMS). The periphery-to-CNS transmission of inflammatory molecules is a major pathophysiological pathway in MS. This could include signalling via extracellular vesicle (EV) microRNAs (miRNAs). In this study, we investigated the serum EV miRNome in CIS and RRMS patients and matched controls, with the aims to identify MS stage-specific differentially expressed miRNAs and investigate their biomarker potential and pathophysiological relevance. miRNA sequencing was conducted on serum EVs from CIS-remission, RRMS-relapse, and viral inflammatory CNS disorder patients, as well as from healthy and hospitalized controls. Differential expression analysis was conducted, followed by predictive power and target-pathway analysis. A moderate number of dysregulated serum EV miRNAs were identified in CIS-remission and RRMS-relapse patients, especially relative to healthy controls. Some of these miRNAs were also differentially expressed between the two MS stages and had biomarker potential for patient-control and CIS–RRMS separations. For the mRNA targets of the RRMS-relapse-specific EV miRNAs, biological processes inherent to MS pathophysiology were identified using in silico analysis. Study findings demonstrate that specific serum EV miRNAs have MS stage-specific biomarker potential and contribute to the identification of potential targets for novel, efficacious therapies.

## 1. Introduction

Multiple sclerosis (MS) is a chronic inflammatory autoimmune disease of the central nervous system (CNS) [1]. Currently, it affects 2.8 million people worldwide [2], with a three-times-higher prevalence in women [3]. In young adults, it is the most common non-traumatic cause of disability [4]. MS aetiology involves various genetic, epigenetic, and environmental factors, that jointly contribute to disease risk [5]. Concerning pathophysiology, the most widely recognized mechanism involves the activation of peripheral self-reactive CD4+ T cells against CNS auto-antigens—including components of the myelin sheath and non-myelin molecules—and the CNS infiltration of these self-reactive cells together with activated B cells and monocytes [1]. This systemic autoimmune activation then causes a sustained immune-inflammatory response in the CNS involving breakdown of the blood–brain barrier (BBB), perivascular demyelination, gliosis, oligodendrocyte injury, and neurodegeneration [6], leading ultimately to overt disease manifestation [7]. Typically, MS onset consists of a first clinical episode of neurological dysfunction suggestive of inflammatory demyelination, termed clinically isolated syndrome (CIS), lasting days or weeks and followed by complete recovery [8]. In 40–60% of cases, CIS converts to clinically definitive MS [9], with 80–85% of patients developing relapsing–remitting MS (RRMS) [10]. RRMS is characterized by alternating periods of acute neurological deficits/relapses and periods of relative clinical stability/remissions [8]. Both CIS and RRMS present with higher inflammatory activity and more frequent waves of immune infiltration into the CNS, which can be visualized by magnetic resonance imaging (MRI), compared to later disease stages [10].

Direct and indirect interactions between peripheral and CNS immune cells, neurons, and glia are inherent to MS pathophysiology. Relatively recent studies have described a novel and potentially relevant mechanism of cell-to-cell communication, namely the transfer of signalling molecules via extracellular vesicles (EVs) [11]. EVs are membrane-enclosed structures secreted by virtually all cell types in the periphery and in the CNS, most abundantly by immune cells [12]. In the parent cell, various molecules are encapsulated into nascent EVs, thereby forming the so-called EV “cargo”; these include cytosolic and membrane proteins, lipids, mRNAs, and high amounts of small non-coding RNAs, including microRNAs (miRNAs) [13]. The molecular loading process is selective, so that the EV cargo is dependent on the status of the parent cell and can be influenced by pathological processes occurring in the tissue of origin [14]. Loaded EVs are then released into the extracellular space and can be transported over short and long distances within body fluids, ultimately transferring functionally active molecules to recipient cells [11,15]. EV involvement in diverse MS-relevant processes, including (auto)antigen presentation, viral infection, BBB breakdown, leukocyte activation and CNS infiltration, and the transfer of pro-inflammatory molecules to and within the CNS, has been described [16,17]. Moreover, higher numbers of circulating EVs have been reported in the periphery and cerebrospinal fluid (CSF) of MS patients compared to healthy controls, as well as during relapse compared to remission in MS patients [18]. Together, these findings are consistent with a pathophysiological role for EVs in MS that necessitates improved understanding.

One recent focus of biomarker and pathophysiology research in MS is miRNAs [17,19]. miRNAs are 18–22-nucleotide-long, non-coding RNAs that exert post-transcriptional regulation, typically via the repression or degradation of their transcriptional (mRNA) targets [20]. A major regulatory involvement of miRNAs has been described in a number of immune system and CNS processes, including some relevant to MS pathophysiology [17,21]. Moreover, several studies have identified changes in miRNA expression in the periphery (blood, plasma, serum, and peripheral blood leukocytes) [22], CSF [23] or brain tissue [19] of MS patients compared to healthy controls (or controls with non-inflammatory neurological disorders in the case of CSF studies). Overall, some 650 miRNAs have been reported as differentially expressed to date, with a 27.5% replication likelihood across at least two independent studies [22]. This limited replicability has been attributed to methodology, e.g., sample type and processing, and to study subjects, e.g., MS stage, within-stage relapse/remission, and control subjects [23]. Accordingly, there has been little use of miRNAs as MS biomarkers to date [22].

The miRNome is more concentrated in EVs than in whole blood [24]; the EV miRNome is stable [25], and EV miRNA expression is modulated strongly by immune-inflammatory processes [26]. Moreover, EVs can enter the CNS by crossing the BBB during inflammation or viral infection [27,28]. In the opposite direction, CNS EVs can be released into the peripheral circulation [29,30,31,32]. Accordingly, specific EV miRNAs may constitute sensitive indicators of systemic inflammation and of CNS immune status. In the last five years, a small number of studies have identified differences in the peripheral EV miRNA profiles of MS patients and healthy control subjects (reviewed in [33]); two studies investigated the entire EV miRNome [34,35]. Whilst the limited number of studies and high between-study heterogeneity of subjects and methods preclude general conclusions, these preliminary findings are promising and warrant further investigation into peripheral EV miRNAs as potential biomarkers for MS.

In this study, we investigated the expression of the serum EV miRNome in CIS-remission patients and RRMS-relapse patients, and compared it with matched controls and between the two MS stages. To identify EV miRNA changes that were MS-related rather than associated with viral inflammatory CNS disorders, a comparison with patients with the latter was also conducted. Our first aim was to investigate whether specific EV miRNAs were differentially expressed at one or the other MS stage. Having identified such EV miRNAs, their potential as candidate biomarkers was quantified, and the biological pathways via which they may contribute to MS pathophysiological mechanisms were investigated. These detailed serum EV miRNome analyses were completed by a preliminary description of the predictive association between the serum and CSF EV miRNomes in RRMS patients.

## 2. Results

### 2.1. Differential Expression Analysis of Serum EV miRNAs

**Figure 1:**
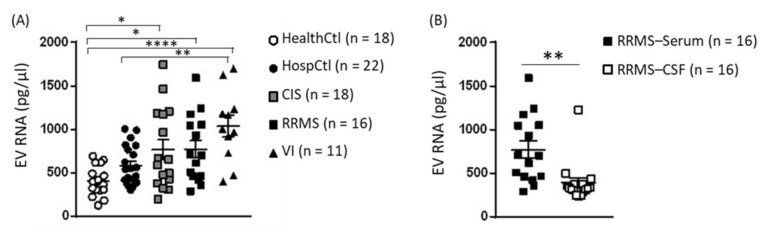
Extracellular vesicle total RNA concentration in serum and CSF samples from healthy controls (HealthCtl), hospitalized controls (HospCtl), CIS-remission patients (CIS), RRMS-relapse patients (RRMS), and viral inflammation patients (VI). Individual values and mean ± standard error of the mean are given. (**A**) Serum EV total RNA concentration. One-way ANOVA of group yielded a significant effect (F(4, 74) = 3.676, *p* = 0.0001). * *p* < 0.05, ** *p* < 0.01, **** *p* < 0.0001, post-hoc Tukey’s test. (**B**) Total RNA concentration of serum and CSF EVs from RRMS patients. ** *p* = 0.007, paired two-tailed Student’s *t*-test.

Before proceeding with small-RNA library preparation, the total RNA concentration of serum and CSF EVs was measured. With respect to serum EV total RNA, this was more concentrated in CIS and RMMS than in HealthCtl and in VI than in HealthCtl and HospCtl (F(4, 74) = 3.676, *p* < 0.0001; Figure 1A). The EV total RNA concentration in serum was higher than in matched CSF samples (t(14) = 3.133, *p* = 0.007; Figure 1B).

**Figure 2:**
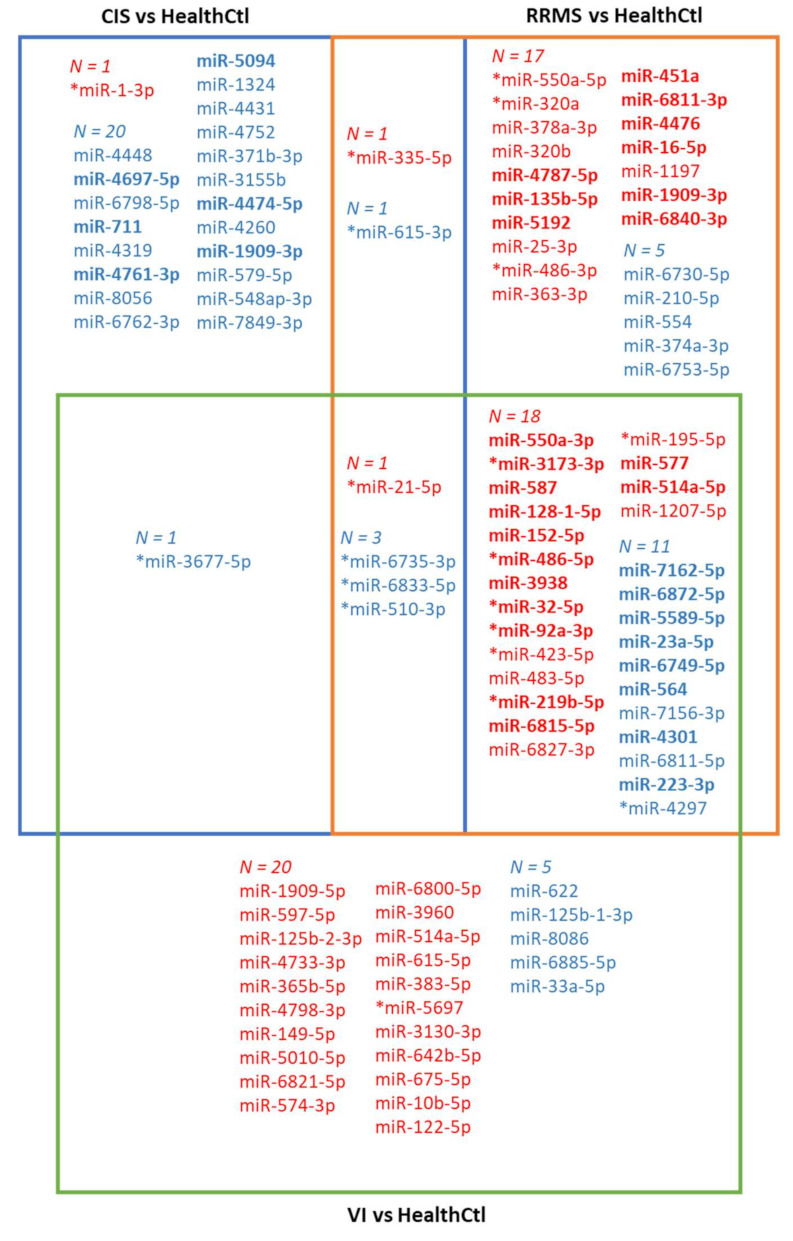
Venn diagram depicting the findings of pair-wise differential expression analysis (DEA, *p* < 0.01, log2 fold change ≥ 1 or ≤ −1) of serum EV miRNomes in different subject groups. As a first step, DEA was conducted for CIS vs. HealthCtl, RRMS vs. HealthCtl, and VI vs. HealthCtl. EV miRNAs indicated in blue were down-regulated in the corresponding disease state(s) relative to HealthCtl, and miRNAs indicated in red were up-regulated in the corresponding disease state(s) relative to HealthCtl. Next, DEA was conducted for CIS vs. RRMS. EV miRNAs indicated in blue and bold were down-regulated in RRMS relative to HealthCtl and CIS, and miRNAs indicated in red and bold were up-regulated in RRMS relative to HealthCtl and CIS. Therefore, miR-4697-5p, miR-711, miR-4761-3p, miR-5094, miR-4474-5p, and miR-1909-3p were CIS-remission-specific relative to HealthCtl and RRMS-relapse, whilst miR-4787-5p, miR-135b-5p, miR-5192, miR-451a, miR-6811-3p, miR-4476, miR-16-5p, miR-1909-3p, and miR-6840-3p were RRMS-relapse-specific relative to HealthCtl and CIS-remission. * EV miRNAs that were also dysregulated, and in the same direction, in the comparison HospCtl relative to HealthCtl.

Per sample, 731-2407 (mean = 1777) mature miRNAs were identified. Following outlier subject exclusion (HealthCtl *n* = 2, HospCtl *n* = 2, CIS *n* = 2), the differential expression analysis of serum EV miRNomes was performed; the findings for the pair-wise comparisons between CIS, RRMS, and VI patients versus HealthCtl are presented in Figure 2. In comparison with HealthCtl, 28 EV miRNAs were identified in CIS patients, 3 up-regulated and 25 down-regulated (four down-regulated miRNAs were also significant after multiple comparison correction; adjusted *p* < 1 × 10^−16^. In RRMS patients, 57 EV miRNAs were identified, 37 up-regulated and 20 down-regulated, (16 up-regulated and 13 down-regulated at adjusted *p* < 1 × 10^−16^ –0.02). In VI patients, 59 EV miRNAs were identified, 39 up-regulated and 20 down-regulated (25 up-regulated and 5 down-regulated at adjusted *p* < 1 × 10^−16^ –0.05). In all three patient groups, miR-21-5p was up-regulated and three miRNAs–miR-6735-3p, miR-6833-5p, and miR-510-3p—were down-regulated, whilst miR-335-5p was up-regulated and miR-615-3p was down-regulated in both CIS and RRMS. Moreover, miR-3677-5p was down-regulated in both CIS and VI, whilst 18 miRNAs were up-regulated and 11 were down-regulated in both RRMS and VI.

Compared to the control group HospCtl (Figure S2), there was differential expression of 20 EV miRNAs (1 up-regulated and 19 down-regulated) in CIS patients, of 27 EV miRNAs (12 up-regulated and 15 down-regulated) in RRMS patients, and of 37 EV miRNAs (27 up-regulated and 10 down-regulated) in VI patients. Of these, two—miR-4751 and miR-141-3p—were down-regulated in all three groups and two—miR-5697 and miR-568—were down-regulated in CIS and RRMS; moreover, miR-122-5p was up-regulated in CIS and VI, and in RRMS and VI, six EV miRNAs were up-regulated—miR-550a-3p, miR-152-5p, miR-128-1-5p, miR-3938, miR-587, and miR-5692a—and nine were down-regulated—miR-7162-5p, miR-4751, miR-564, miR-141-3p, miR-4301, miR-6749-5p, miR-4320, miR-33a-5p, and miR-1-3p.

Of the 25 EV miRNAs that were down-regulated in CIS compared to HealthCtl, 6—miR-4697-5p, miR-711, miR-4761-3p, miR-5094, miR-4474-5p, and miR-1909-3p—were also down-regulated compared to RRMS and designated as CIS-specific. Of the 37 EV miRNAs that were up-regulated in RRMS compared to HealthCtl, 9—miR-4787-5p, miR-135b-5p, miR-451a, miR-6811-3p, miR-4476, miR-16-5p, miR-6840-3p, and miR-1909-3p—were also up-regulated compared to CIS and designated as RRMS-specific (Figure 2). Three of the six CIS-specific miRNAs—miR-4697-5p, miR-47474, and miR-1909-3p—were also down-regulated compared to HospCtl, and the RRMS-specific miR-1909-3p was also up-regulated compared to HospCtl. Of note, miR-1909-3p was significantly down-regulated in CIS versus both HealthCtl and HospCtl, significantly up-regulated in RRMS versus both HealthCtl and HospCtl, and significantly up-regulated in RRMS versus CIS. Subsequent analyses were conducted with the CIS-specific and RRMS-specific dysregulated serum EV miRNAs.

Comparing the two control groups with each other showed that 21 EV miRNAs were dysregulated in HospCtl compared to HealthCtl, 15 up-regulated and 6 down-regulated (3 and 5, respectively, after correction for multiple comparison). Of these dysregulated miRNAs, 12 and 5 were also in the up- and down-regulated groups in RRMS patients compared to HealthCtl, and 3 and 5 were also in the up- and down-regulated groups in CIS patients compared with HealthCtl (Figure 2). Therefore, these EV miRNAs were up- or down-regulated in non-inflammatory neurological patients and not specifically in RRMS or CIS patients.

RT-qPCR was conducted for two RRMS-specific up-regulated miRNAs—miR-16-5p and miR-451a. miR-16-5p was found to be up-regulated in RRMS (mean Delta Cq of 3.88) compared with CIS (mean Delta Cq of 5.16) patients (t(27) = 5.18, *p* < 0.0001) when using RT-qPCR (Figure S3A). miR-451a was not shown to be significantly up-regulated when using RT-qPCR (CIS: 3.0; RRMS: 2.7; *p* = 0.32; Figure S3B). In the case of both miRNAs, there was a significant between-subject negative correlation between miRNA-Seq normalized counts and Delta Cq values, which was calculated separately for in each of the CIS and RRMS patient sample groups (Table S1; Figure S3C–F).

### 2.2. Predictive Power Analysis of MS Stage-Specific Dysregulated miRNAs

To assess the predictive power of the CIS-specific and RRMS-specific miRNAs, ROC analysis was conducted with z-scored normalized counts of each individual miRNA. Of the six CIS-specific miRNAs, five had significant predictive power for CIS–HealthCtl separation, with an area under the curve (AUC) = 0.78–0.86 and *p* ≤ 0.007. These miRNAs, and including miR-1909-3p, also yielded significant predictive power for CIS–RRMS separation, with an AUC = 0.84–0.90 and *p* ≤ 0.001. Of the nine RRMS-specific miRNAs, eight had significant predictive power for RRMS–HealthCtl separation, with an AUC = 0.74–0.88 and *p* ≤ 0.02. These same miRNAs, except for miR-6811-3p, also yielded significant predictive power for RRMS–CIS separation, with AUC = 0.71–0.91 and *p* ≤ 0.04.

**Figure 3:**
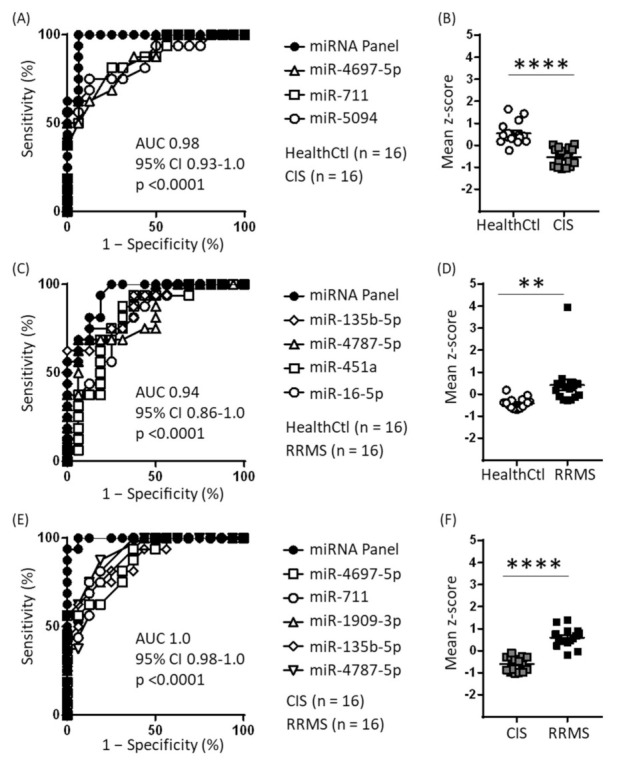
Receiver operating characteristic (ROC) analysis of CIS-specific and RRMS-specific EV miRNAs. (**A**) Using z-score transformation of normalized counts, 5 of the 6 CIS-specific down-regulated miRNAs were found to have significant predictive power for CIS–HealthCtl separation, and the mean of the three most predictive miRNAs yielded a profile with even greater predictive power. (**B**) Individual subject means of the z scores for the three miRNAs given in (A). **** *p* < 0.0001, unpaired two-tailed Student’s *t* test. (**C**) Using z-score transformation of normalized counts, 8 of the 9 RRMS-specific up-regulated miRNAs were found to have significant predictive power for RRMS–HealthCtl separation, and the mean of the four most predictive miRNAs yielded a profile with even greater predictive power. (**D**) Individual subject means of the z scores for the four miRNAs given in C. ** *p* = 0.003, unpaired two-tailed Student’s *t* test. (**E**) Using z-score transformation of normalized counts, all 6 CIS-specific down-regulated miRNAs and 7 of 9 RRMS-specific up-regulated miRNAs were found to have significant predictive power for CIS–RRMS separation, and the mean of the three most predictive CIS-specific and three most predictive RRMS-specific miRNAs yielded a profile with even greater predictive power. (**F**) Individual subject means of the z scores for the six miRNAs given in E. **** *p* < 0.0001, unpaired two-tailed Student’s *t* test.

As a next step, the CIS- or RRMS-specific miRNAs were ranked based on their ROC AUC and *p* values, and panels comprising these miRNAs were investigated with respect to their predictive power relative to individual miRNAs. With respect to CIS–HealthCtl separation, a panel comprising the three most predictive miRNAs—miR-4697-5p, miR-711, and miR-5094—had higher predictive value than the most predictive single miRNA (Figure 3A), whilst the inclusion of additional CIS-specific down-regulated miRNAs did not yield further increase. Furthermore, the per subject mean z-scores of these three miRNAs were lower in CIS than HealthCtl (t(30) = 6.62, *p* < 0.0001; Figure 3B). For RRMS–HealthCtl separation, a panel comprising the four most predictive miRNAs—miR-135b-5p, miR-4787-5p, miR-451a, and miR-16-5p—had higher predictive value than the most predictive miRNA (Figure 3C), whilst the inclusion of additional RRMS-specific up-regulated miRNAs did not yield further increase. Furthermore, the per subject mean z-scores of these four miRNAs was higher in RRMS than HealthCtl (t(30) = 3.24, *p* = 0.003; Figure 3D). For CIS–RRMS separation, a panel comprising the three most predictive CIS-specific down-regulated miRNAs—miR-1909-3p, miR-711, and miR-4697-5p—and the three most predictive RRMS-specific up-regulated miRNAs—miR-4787-5p, miR-1909-3p, and miR-135b-5p—yielded higher predictive power than any of the individual CIS- or RRMS-specific miRNAs (Figure 3E); the per subject mean z-scores of these five miRNAs were higher in RRMS compared to CIS (t(30) = 8.95, *p* < 0.0001; Figure 3F). For the CIS–RRMS panel, between-subject correlation analyses between mean miRNA z-scores and EDSS scores were conducted separately for CIS and RRMS patients (Figure S4A,B): for CIS patients, a positive correlation was identified (r = 0.78, 95% CI = 0.44–0.92, *p* = 0.0007), whilst for RRMS patients, there was no correlation (r = −0.24, *p* = 0.36).

### 2.3. Target Mining and Pathway Analysis of Disease Stage-Specific Dysregulated miRNAs

The target mining and pathway analyses of CIS- and RRMS-specific EV miRNAs were conducted to obtain insights into their biological functions and their potential relevance to the pathophysiology of these two MS stages. For the 6 CIS-specific down-regulated EV miRNAs, 89 validated target transcripts were identified. The over-representation analysis of these transcripts did not yield any significant pathway in the Reactome Pathway Database. For the 9 RRMS-specific EV miRNAs, 479 validated target transcripts were identified. The over-representation analysis of these transcripts identified 36 pathways involving 222 of the target transcripts in the Reactome Pathway Database. Table 1 presents the pathways ranked 1–5 based on adjusted *p* values, and a complete list of the significant pathways is given in Table S2. Regarding pathway functional annotation, some of the particularly interesting examples included 10 signal transduction pathways (including Ca2+ signalling, Wnt signalling, MAPK family signalling, TGFB-related signalling, and PTEN regulation), 7 immune system pathways (including innate immune system, adaptive immune system, and cytokine signalling pathways, including signalling by interleukins, neutrophil degradation, and MHC-I-mediated antigen processing and presentation), and 7 vesicle-mediated transport pathways (including membrane trafficking, Rab-mediated trafficking, and Golgi-to-ER transport).

### 2.4. Serum and CSF EV miRNA Expression in RRMS Patients

**Figure 4:**
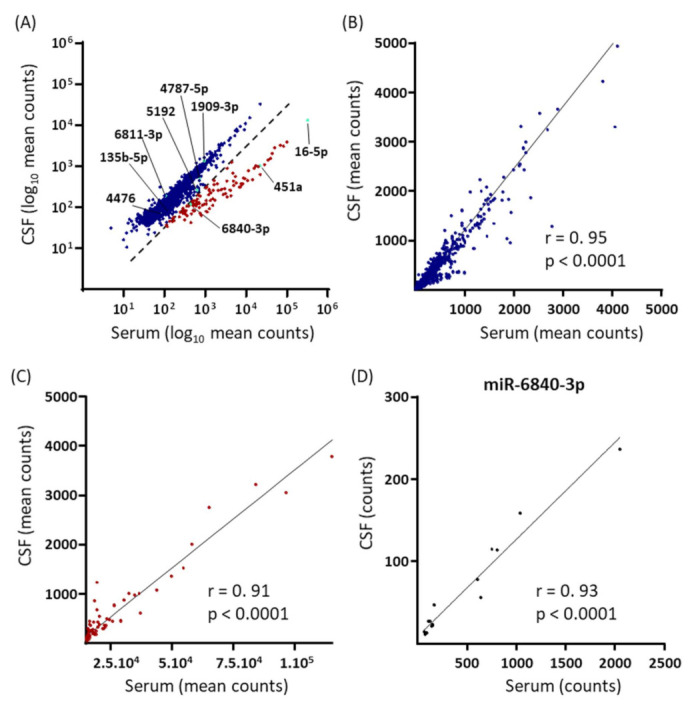
Comparison of EV miRNome expression in matched serum and CSF samples collected from RRMS-relapse patients (*n* = 16). (**A**) Scatterplot of the log10 transformed mean normalized counts for each of the 2151 mature EV miRNAs quantified in both sample compartments. Two miRNA populations were visualized based on their serum–CSF associations. The dashed line indicates the qualitative best-fit-line of separation between the two miRNA populations. In the larger population, with data points depicted in blue, serum and CSF expression levels were similar. In the smaller population, with data points depicted in red, serum expression levels were high relative to CSF levels. The nine RRMS-relapse specific miRNAs are indicated in turquoise and identified. (**B**) Pearson’s product-moment correlation analysis of the linear normalized counts of serum vs. CSF miRNAs belonging to the larger miRNA population in A; 12 miRNAs lay outside the axis limits. (**C**) Pearson’s correlation analysis of the linear normalized counts of serum vs. CSF miRNAs belonging to the smaller miRNA population in A; 1 miRNA lays outside the axis limits. (**D**) Pearson’s correlation analysis of serum vs. CSF linear normalized counts for the RRMS-specific miR-6840-3p. Pearson’s correlation coefficients and *p* values are reported in the graph.

As a preliminary investigation into the extent of the predictive association between EV miRNome expression in matched serum and CSF samples from RRMS patients, the log-transformed sample-mean expression values (log10 of normalized counts) of all 2151 miRNAs identified in both compartments were plotted (Figure 4A). Two miRNA populations were identified qualitatively. One included the majority of the identified miRNAs (*n* = 2038) and consisted of miRNAs of which the expression levels were similar in serum and CSF and relatively low in both compartments; six of the nine RRMS-specific miRNAs belonged to this population, including two of the four miRNAs composing the biomarker panel for RRMS-relapse. The second, smaller cluster (*n* = 128) consisted of miRNAs of which the expression levels were markedly higher in serum than in CSF and included miRNAs with the highest serum expression levels; three of the nine RRMS-specific miRNAs belonged to this population, including two of the four miRNAs composing the biomarker panel for RRMS-relapse. For each population, Spearman’s rank-order correlations were then calculated for the mean linear miRNA expression values in the two compartments. For the large population, there was a significant positive correlation (r = 0.95, 95% CI 0.94–0.95 *p* < 0.0001; Figure 4B), and this was also the case for the small population (r = 0.91, 95% CI 0.87–0.94 *p* < 0.0001; Figure 4C). Finally, Pearson product-moment correlations between serum and CSF EV miRNA expression were calculated for the nine up-regulated RRMS-specific serum miRNAs: miR-6840-3p demonstrated a significant positive correlation (r = 0.93, 95% CI 0.80–0.98, *p* < 0.0001; Figure 4D), whilst the other eight did not (r < 0.33, *p* ≥ 0.2).

## 3. Discussion

In the present study, we investigated the serum EV miRNome of CIS-remission and RRMS-relapse patients compared with control subjects, healthy or hospitalized, and with viral inflammatory CNS disorder patients as a positive control. Extracellular vesicles provide a more enriched source of miRNAs than whole blood [24], their miRNAs are more stable and less affected by pre-analytical variables [25], CNS-derived EV miRNAs in blood can inform on CNS status, and EVs transfer functionally active miRNAs to recipient cells [15,36], including from peripheral immune cells to CNS cells [27]. The aims of the present study were to assess whether MS stage-specific EV miRNAs could be identified and, if so, to investigate their predictive power as biomarker candidates and their putative regulatory involvement in biological pathways that could contribute to MS pathophysiology. A small number of serum EV miRNAs were down-regulated in CIS-remission patients relative to controls and RRMS-relapse patients, whilst a small number of serum EV miRNAs were up-regulated in RRMS-relapse patients relative to controls (especially healthy controls) and CIS-remission patients. That is, there was a double dissociation in the consistent changes occurring in the EV miRNome at the two MS stages. Among the dysregulated miRNAs, some had significant predictive power and therefore biomarker potential for each of the two MS stages, particularly when aggregated into panels of 3-4 miRNAs. Moreover, the pathway analysis of the transcriptional targets of the RRMS-specific up-regulated miRNAs revealed enrichment for pathways of direct relevance to MS pathophysiology.

The proportion of serum EV miRNAs that met the threshold for differential regulation was, at both MS stages, moderate relative to the total miRNAs identified. It is noteworthy that in CIS-remission patients, the majority of these miRNAs were down-regulated relative to controls, whilst in RRMS-relapse patients, the majority were up-regulated relative to controls. Furthermore, it is important that some EV miRNAs in CIS-remission patients were down-regulated relative to controls and RRMS-relapse and that some EV miRNAs in RRMS-relapse patients were up-regulated relative to controls and CIS-remission patients. Furthermore, the CIS-dysregulated EV miRNAs showed negligible overlap with VI dysregulation, whilst there was extensive overlap between RRMS and VI dysregulation. For both MS stages, the number of dysregulated miRNAs was lower compared to hospitalized patients than compared to healthy controls, which could reflect the higher inflammatory status of EV-synthetizing cells in the former and highlights the importance of control subject selection in such studies.

At the level of specific EV miRNA identity, a comparison of the differentially expressed EV miRNAs in this study with those reported for non-EV-specific miRNAs in studies focusing primarily on RRMS (reviewed in [19,22,23]) identified only a small number of overlaps. Examples include the up-regulation of miR-320a/b and miR-25-3p (RRMS but not RRMS-specific in present study) and the down-regulation of miR-579 and miR-548a-3p (CIS but not CIS-specific in present study). With respect to previous studies of peripheral EV miRNAs in MS, of which nine have been conducted to date to the best of our knowledge, up-regulated miR-451a in RRMS (RRMS-specific in the present study) is consistent with the work of Ebrahimkhani et al. [34] and Manna et al. [37], with the latter reporting the normalising of EV miR-451a by IFN-β treatment; up-regulated EV miR-25-3p was RRMS-specific in the present study and was previously reported to be up-regulated in RRMS in plasma EVs [38]. Some miRNAs previously reported to be differentially expressed in MS patients in non-EV-specific samples (e.g., miR-21 and miR-23a) and in EV samples (e.g., miR-122 and miR-92a-3p) were dysregulated in both RRMS and VI patients in our study, thereby highlighting the importance of including CNS inflammatory disorder patient samples as a positive control. Additionally, this study appears to be the first to investigate the CIS EV miRNome; four studies have assessed the CIS non-EV miRNA profile, one in whole blood [39] and three in CSF [40,41,42].

Six serum EV miRNAs were down-regulated in CIS compared to HealthCtl and RRMS (CIS-remission-specific), and nine serum EV miRNAs were up-regulated in RRMS compared to HealthCtl and CIS (RRMS-relapse-specific). These MS stage-specific EV miRNAs had significant predictive power with respect to MS stage versus healthy and CIS-remission versus RRMS-relapse demarcation. This was particularly the case when the normalized expression values of 3–4 of these predictive serum EV miRNAs were pooled to provide a biomarker panel. The EV miRNA panel identified for CIS–RRMS separation was partly different from the patient-control panels and had the highest predictive power. Interestingly, there was a positive correlation between the mean z-score of the miRNAs in the CIS–RRMS—which were up-regulated in RRMS compared to CIS—and the EDSS scores of CIS patients. These findings are consistent with a potential for EV miRNAs to function as MS stage-specific biomarker panels and indicate a certain correlation between EV miRNA expression levels and degree of disability, at least during the earliest phase of the disease. Of note, some non-EV-specific CSF miRNAs have also been reported to distinguish between CIS and clinically definitive MS, as well as to be predictive of CIS-to-MS conversion [42].

The CIS-specific and RRMS-specific EV miRNAs were then investigated with respect to their transcriptional targets and, on the basis of these, the biological pathways they are predicted to act upon. For CIS-specific down-regulated EV miRNAs, there were 89 validated targets, but the identities of these were such that they did not significantly predict any biological pathway, suggesting that these miRNAs are heterogeneous with respect to target transcripts/biological pathways. Notwithstanding, it is pertinent to point out that the down-regulated CIS-specific miR-4697 has been reported to target the signal transducer and activation of transcription (STAT) 1 mRNA [43]. In CIS-remission, the down-regulation of EV miR-4697 may enable more STAT1 translation and the subsequent STAT1-mediated suppression of Th17 cells [44], which would be significant given the latter’s major role in MS pathophysiology [1]. For RRMS-specific up-regulated EV miRNAs, there were 479 validated transcriptional targets, and these significantly predicted 36 biological pathways. This suggests that up-regulated EV miRNAs play an orchestrated regulatory function in various processes during MS relapse. Among those identified were immune system pathways including innate and adaptive immune system and cytokine-mediated signalling. The over-activation of the immune system and increased cytokine production are cardinal features of MS, and they are especially heightened during relapse [10]. Identified signal transduction pathways included TGFB-mediated signalling and PTEN regulation, both of which have been reported as targets of dysregulated non-EV-specific miRNAs in MS [19,22]. Several Wnt signalling-related pathways were also identified, the dysregulation of which has been implicated in BBB breakdown [45] and the impairment of axonal remyelination [46]. Moreover, we identified cell cycle pathways, which have often been reported in MS target-pathway studies of dysregulated non-EV-specific miRNAs and associated with the failure of mechanisms controlling immune cell proliferation [22]. The auto-proliferation of peripheral auto-reactive T cells is increased in MS patients [47], more during remission than relapse presumably because of the increased transfer of these cells to the CNS during the latter [48]. Here, the up-regulation of miRNAs targeting genes involved in cell cycle pathways specifically in RRMS-relapse patients could suggest a role for EV miRNAs in inhibiting the peripheral proliferation of pathogenic immune cells in favour of their CNS transmigration. Vesicular transport pathways, including those related to endocytic transport, were also over-represented; the biogenesis and release of an EV subpopulation relies on the endocytic pathway [11]. Interestingly, the validated targets of the RRMS-relapse-specific miRNAs did not include some of the genes most implicated in MS pathophysiology, e.g., *IFNG*, *TNFR1*, *IL7R*, and *IL2RA* [4]. This could indicate that EV miRNAs regulate genes with subsidiary roles in disease mechanisms rather than those involved in the main pathogenic pathways; however, it could also reflect the relative paucity of functional studies on the role of miRNA-mediated gene regulation in MS. Together, these findings indicate that RRMS-relapse is associated with the up-regulation of serum EV miRNAs that target mRNAs involved in some of the major cellular and molecular pathways underlying MS relapses, and these pathways partially overlap with those identified in MS non-EV-specific miRNA studies.

Of course, understanding the role of EV miRNAs in the pathophysiology of MS will require detailed study of the CSF in addition to blood EV miRNome. In this regard, the present study also included a comparison of the two compartments in the RRMS patient subjects. Two main populations of miRNAs were identified based on their serum–CSF associations: one included the majority of all identified miRNAs and had similar expression levels in the two sample compartments; the second population was smaller and consisted of miRNAs with high absolute serum expression levels and higher expression levels in serum relative to CSF. For both these miRNA populations, there was an overall positive correlation between their mean serum and CSF expressions. However, when the nine RRMS-specific EV miRNAs were individually analysed, a significant (positive) correlation was only observed for miR-6840-3p, suggesting either the differential regulation of specific serum and CSF EV miRNAs during relapse or the preferential transport of the latter towards the periphery or the CNS.

In summary, the present study contributes to the currently small number of studies of the EV miRNome in MS. In the serum EV miRNome of CIS-remission and RRMS-relapse patients, a number of dysregulated EV miRNAs was identified relative to healthy controls, and we were particularly interested in identifying those that were also dysregulated relative to the other disease stage. This double-dissociation analysis identified six CIS-remission-specific down-regulated EV miRNAs and nine RRMS-relapse-specific up-regulated EV miRNAs. These EV miRNAs were efficacious as biomarkers, particularly when pooled, with respect to patient-control and CIS–RRMS separation. Further replication studies in independent and larger cohorts are required to confirm the presence and identity of serum EV miRNAs with biomarker potential for MS. Moreover, analyses of mRNA targets and biological pathways of the RRMS-specific EV miRNAs identified cellular and molecular processes highly relevant to MS pathophysiology, thereby highlighting the importance of future mechanistic studies of the EV miRNome in MS in order to elucidate its aetio-pathophysiology and to identify potential targets for novel, efficacious therapies.

## 4. Materials and Methods

### 4.1. Participants

The study was approved by the Ethics Committee of the University of Ulm (Ulm, Germany; vote and approval number 20/10), and all participants provided written informed consent. All patients attended the Neurology Clinic, University of Ulm (Ulm, Germany). The demographic and clinical characteristics of the study subjects are summarized in Table 2. CIS patients (*n* = 18) and RRMS patients (*n* = 16) were diagnosed according to the revised McDonald criteria [49], and half of the CIS patients went on to develop clinically definitive MS within 12–80 months (mean 30.8) from sample collection. All CIS serum samples except one were collected after CIS waning, i.e., during remission, and all samples except one were collected prior to onset of any disease-modifying therapy (DMT). RRMS patient samples were collected during relapse, and, with one exception, patients were not receiving DMT at the time of sample collection. The clinical disability of CIS and of RRMS patients was assessed using the Expanded Disability Status Scale (EDSS), on which average scores were 1.7 and 2.3, respectively. As a positive control, samples were also collected from patients with a diagnosis of a viral inflammatory CNS disorder (VI, *n* = 11), namely viral meningitis, viral encephalitis, or viral meningoencephalitis. Two different control-subject groups were included. A healthy control group (HealthCtl, *n* = 18) was recruited from the community via advertisement, and a hospitalized control group (HospCtl, *n* = 22) comprised patients at the Neurology Clinic, University of Ulm who were examined to exclude an inflammatory process of the CNS and received a differential diagnosis, including migraine, depression, or granulomatosis; this control group enabled the identification of MS-specific EV miRNAs compared to non-inflammatory neurological patients.

Blood samples were collected in 7.5 mL serum collection tubes (Sarstedt AG & Co. KG, Nümbrecht, Germany), left at room temperature (RT) for 10 min to allow for blood clotting, and then centrifuged at 2000× *g* and RT for 10 min. From the RRMS patients specifically, at the same consultation, a sample of cerebrospinal fluid (CSF) was collected via lumbar puncture according to a standardized protocol [50] and centrifuged at 2000× *g* and RT for 10 min. Serum and CSF aliquots of 200 µL were transferred into 0.5 mL microcentrifuge cryotubes and stored immediately at −80 °C until further processing.

### 4.2. EV Isolation and RNA Extraction

Extracellular vesicles were isolated from one 200 µL serum aliquot per subject using size exclusion chromatography (SEC) [51]. Briefly, samples were thawed at 37 °C and centrifuged at 3000× *g* and RT for 10 min. The supernatant (170 µL) was applied onto an SEC column (qEV single 35 nm, Izon Science Ltd., Oxford, UK) and eluted by the progressive addition of freshly filtered phosphate buffered saline (PBS), which allowed for the separation and collection of five distinct 200 µL fractions containing EVs. CSF samples were thawed at RT and centrifuged at 3000× *g* and RT for 1 min. The supernatant (100–200 µL) was applied onto 1–2 SEC columns (100 µL per column), and EVs were collected as described for serum samples. The presence of EVs and the absence of contaminants, most notably low-density lipoproteins, in the purported EV-rich fractions were validated using several complementary methods in accordance with the Guidelines of the International Society for Extracellular Vesicles [52]. These methods were tuneable resistive pulse sensing, transmission electron microscopy, Western blotting, and LDL-cholesterol assay, details for which are given in Supplementary Information (including Figure S1A–I).

Per sample, EV-rich fractions were pooled and then ultra-filtrated to a volume of 200 µL using an Amicon Ultra-4 10K Centrifugal Filter Device (Merck Millipore Ltd., Cork, Ireland), to which 600 µL of lysis buffer (Norgen Biotek Corp., Thorold, ON, Canada) containing 1% β-mercaptoethanol were added. Lysed samples were immediately frozen on dry ice and stored at −80 °C until RNA extraction. RNA was extracted using the Plasma/Serum Purification Mini Kit (Norgen Biotek Corp., Thorold, ON, Canada; cat. 55000) according to the manufacturer’s instructions (title of instruction protocol: Exosomal RNA Purification from Exosomes Already Purified via Ultracentrifugation, Exoquick, Filtration or any other Precipitation Method). RNA concentration and size distribution were assessed using the Agilent RNA 6000 Pico kit and a 2100 Bioanalyzer system (Agilent Technologies AG, Waldbronn, Germany). RNA samples were stored at −80 °C until library preparation.

### 4.3. MicroRNA Sequencing

Small-RNA libraries for multiplexed sequencing were prepared from the EV RNA samples using the QIAseq miRNA Library kit (Qiagen Sciences Inc., Germantown, MD, USA; cat. 331505) according to the manufacturer’s instructions. Library quality was assessed using a TapeStation High Sensitivity DNA system (Agilent Biotechnologies AG, Waldbronn, Germany). The cDNA libraries were pooled using an equal amount of cDNA per sample library, and sequencing was conducted on an Illumina NovaSeq 6000 System (Illumina Inc., San Diego, CA, USA) with a sequencing depth of 5 million reads per sample and a single-end 100 bp read configuration.

### 4.4. Bioinformatics Analysis

The raw sequencing data were uploaded onto the GeneGlobe Data Analysis Center (Qiagen Sciences Inc., https://geneglobe.qiagen.com/us/analyze) for pre-processing and quantification. First, 3′ adapters and low-quality bases were trimmed using Cutadapt. Next, the insert sequences and unique molecular identifiers (UMIs) were identified. Read mapping was performed according to a sequential alignment strategy using Bowtie: Firstly, reads were mapped to miRBase mature, miRBase hairpin, non-coding RNA, mRNA, and other RNA databases in order to identify perfect matches. Second, mapping to a species-specific miRBase mature database was conducted (1–2 mismatches tolerated). Third, all remaining unmapped sequences were aligned to the human genome (Genome Reference Consortium GRCh38) to identify possible novel miRNA molecules. All reads assigned to each specific miRNA were quantified, and the associated UMIs were aggregated to count unique molecules. To assess whether any systematic amplification bias was introduced during library preparation, Spearman’s rank-order correlation analysis of total read counts versus total UMIs was conducted; high correlations (rho = 0.99–1) demonstrated an absence of bias.

Downstream expression analyses were conducted in R. miRNAs with less than 10 counts in ≥50% of the samples were considered to be background noise and discarded. Spearman’s rank-order correlations were calculated for each subject’s miRNA counts versus the group mean miRNA counts in order to assess within-group homogeneity and identify possible outliers. Subjects for whom correlation coefficients were below the 25th percentile of all subjects’ correlation coefficients per group were considered outliers and excluded from the downstream analysis; this applied to two HealthCtl subjects, two HospCtl subjects, and two CIS subjects (including the only CIS subject receiving DMT at sample collection). miRNome differential expression analysis was implemented using the Bioconductor 3.14 package EdgeR [53]. Firstly, aggregate counts were normalized to the trimmed mean of the M values (TMM), and then differential expression analysis was conducted using the quasi-likelihood F-test. The criteria thresholds for differential expression were *p* value < 0.01 and log2 fold change ≥1 or ≤−1. In addition, adjusted *p* values were calculated using the false discovery rate according to the Benjamini–Hochberg method. For serum EV miRNomes, the following pair-wise differential expression analyses were conducted: CIS vs. HealthCtl, CIS vs. HospCtl, RRMS vs. HealthCtl, RRMS vs. HospCtl, VI vs. HealthCtl, VI vs. HospCtl, and RRMS vs. CIS. After identifying those miRNAs that were differentially expressed in CIS vs. HealthCtl and CIS vs. RRMS (“CIS-specific”) or in RRMS vs. HealthCtl and CIS vs. RRMS (“RRMS-specific”), they were further investigated with respect to biomarker potential and target-gene pathways.

### 4.5. RT-qPCR

The expression levels of two RRMS-specific up-regulated EV miRNAs—miR-16-5p and miR-451a—were validated using quantitative reverse transcription PCR (RT-qPCR). These two miRNAs were selected because the primers (Qiagen Sciences Inc., Germantown, MD, USA) could be validated in-house with respect to sensitivity and specificity; the primers for the other dysregulated EV miRNAs could not be validated in-house. Per sample, 5 µL RNA (circa 700 pg/µL) were used for reverse transcription, which was conducted using the miRCURY LNA RT kit (Qiagen Sciences Inc., Germantown, MD, USA; cat. 339340) according to the manufacturer’s instructions. A synthetic spike-in miRNA, UniSp6 (Qiagen Sciences Inc., Germantown, MD, USA), was added to the solution to normalize any potential differences in reaction efficiency between samples. The cDNA samples were stored at −20 °C until further processing. The qPCR reaction was prepared using the miRCURY LNA SYBR PCR Kit and miRNA-specific primers (Qiagen Sciences Inc., Germantown, MD, USA) and run using a C1000 Touch Thermal Cycler and a CFX384 Touch Real-Time PCR Detection System (Bio-Rad Laboratories Inc, Hercules, CA, USA); samples were run in triplicate for each miRNA and in duplicate for Unisp6. Data were analysed using CFX Maestro software (2017, Bio-Rad Laboratories Inc, Hercules, CA, USA), and the quantile normalization approach [54] was implemented to identify outlier technical replicates. For statistical analysis, normalized quantification cycle values (referred to as Delta Cq), which were calculated as the difference between the mean Cq of the target miRNA and the Cq of Unisp6, were used. Unpaired two-tailed Student’s *t*-tests were conducted with the Delta Cq values for CIS versus RRMS, and Spearman’s rank-order correlation analyses between normalized miRNA-Seq counts and delta Cq values were conducted for each of the two miRNAs of interest separately within CIS and RRMS subject groups. Statistical analyses were conducted using GraphPad Prism v. 7.

### 4.6. Target-Gene Pathway Analysis

The transcriptional targets of the CIS-specific and RRMS-specific serum EV miRNAs were retrieved from the miRWalk database v. 3.0 [55] using the “miRTarBase” filter function to only select experimentally validated targets [56] and specifically considering interactions between miRNAs and mRNA 3′ UTR. Over-representation analysis was separately performed for the validated targets of CIS-specific and RRMS-specific miRNAs using the Molecular Signatures Database v. 7.4 [57,58]; pathways were retrieved from the Reactome Pathway Database [59]. Significantly enriched pathways were defined as those with a *p* value < 0.01 following Benjamini–Hochberg method adjustment and involving three or more miRNAs.

### 4.7. Statistical Analysis

All statistical analyses were conducted using GraphPad Prism v. 7. The distribution of the data for serum EV total RNA concentration was assessed using a D’Agostino and Pearson normality test; as the data passed the normality test, serum EV total RNA concentration was compared between groups using a one-way analysis of variance (ANOVA), with a significant main effect analysed using post hoc Tukey’s test. A comparison of total EV RNA in serum and CSF samples from RRMS patients was conducted using a paired two-tailed Student’s *t*-test. Receiver-operating characteristic (ROC) analysis was conducted with the same double differentially expressed miRNAs (i.e., CIS- or RRMS-specific) on which target-gene pathway analysis was conducted. Firstly, the miRNA expression values (normalized counts) were z-scored to control for absolute differences in the expression values between miRNAs. For each miRNA differentially expressed in CIS versus HealthCtl and RRMS or in RRMS versus HealthCtl and CIS, the area under the ROC curve (AUC) and corresponding *p* values were calculated as a measure of its predictive power as a biomarker. Then, starting with the two miRNAs with the highest predictive powers based on their AUC and *p* values, each subject’s z-scores for two or more miRNAs were averaged and ROC analysis was conducted with these averaged z-scores to determine whether a miRNA panel could be identified with higher predictive power than that of the most predictive individual miRNA. Furthermore, the means of the z-scores of the miRNAs contributing to the miRNA panels were compared using an unpaired two-tailed Student’s *t*-test. Correlation analysis between the mean z-score of the miRNA panel for CIS–RRMS separation and the EDSS scores was also performed separately for CIS and RRMS subjects; Pearson’s product-moment correlation was used with the CIS dataset because it passed the D’Agostino and Pearson normality test, whilst Spearman’s rank-order correlation was used with the RRMS dataset because it did not pass the normality test. To investigate the predictive association of EV miRNome expression between matched CSF and serum samples from RRMS patients, the log10 of mean normalized counts for all miRNAs identified in both sample compartments was plotted: two miRNA populations were qualitatively identified based on their clustering, and, for each of these, between-compartment Spearman’s correlation was used for mean miRNA linear expression values (data did not pass D’Agostino and Pearson normality test). Finally, between-subject Spearman’s correlation was used on serum versus CSF linear expression values for each of the RRMS-specific miRNAs.

## Figures and Tables

**Table 1:** Five highest over-represented pathways ^1^ identified for validated target genes of the RRMS-specific miRNAs.

Pathway	Adjusted *p* Value	miRNA	Target Transcripts
Vesicle-mediated transport	2.41 × 10^8^	miR-16-5p	*RAB9B*, *CAPZA2*, *DCTN5*, *GALNT1*, *RAB30*, *PPP6R3*, *RAB3IP*, *SBF1*, *TBC1D14*, *KIF2A*, *RACGAP1*, *KIF3B*, *KIF1B*, *TFRC*, *COL4A1*
		miR-6840-3p	*TRAPPC2*, *ACTR1A*, *FZD4*, *GNS*, *TBC1D24*, *GJC1*, *APOL1*
		miR-6811-3p	*RAB13*, *RAB30*, *PAFAH1B2*, *DENND6B*
		miR-5192	*CBL*, *CSNK1D*, *TBC1D13*, *SYT2*, *CALR*
		miR-4476	*RAB5B*, *ACTR2*, *PACSIN1*
		miR-135b-5p	*RAB3GAP2*
Membrane trafficking	2.96 × 10^8^	miR-6840-3p	*TRAPPC2*, *ACTR1A*, *FZD4*, *GNS*, *TBC1D24*, *GJC1*
		miR-6811-3p	*RAB13*, *RAB30*, *PAFAH1B2*, *DENND6B*
		miR-5192	*CBL*, *CSNK1D*, *TBC1D13*, *SYT2*
		miR-4476	*RAB5B*, *ACTR2*, *PACSIN1*
		miR-135b-5p	*RAB3GAP2*
Post-translational protein modification	1.87 × 10^7^	miR-16-5p	*RAB9B*, *CAPZA2*, *DCTN5*, *GALNT1*, *RAB30*, *PPP6R3*, *BIRC5*, *MAP3K7*, *SOCS2*, *NUP155*, *NCOR2*, *BRCA1*, *CUL3*, *FBXL20*, *B3GNT2*, *RAB23*, *GALNT7*, *ASXL1*
		miR-6840-3p	*TRAPPC2*, *ACTR1A*, *APOL1*, *MLEC*, *RCE1*, *SUMF2*, *GGCX*, *DCAF10*, *FOXK1*, *CPM*
		miR-4476	*RAB5B*, *PSMD11*, *PHC2*, *TFAP2A*, *USP12*, *IGFBP4*
		miR-1909-3p	*CANX*, *TFAP2B*, *SENP5*, *DPH2*, *WAC*
		miR-5192	*CSNK1D*, *CALR*, *ST6GAL2*, *RECK*
		miR-6811-3p	*RAB13*, *RAB30*, *KLHL21*, *PEX2*
		miR-135b-5p	*THBS2*
Signalling by interleukins	1.87 × 10^7^	miR-16-5p	*BIRC5*, *MAP3K7*, *SOCS2*, *VEGFA*, *RPS6KA3*, *UBE2V1*, *BCL2*, *IRAK3*, *PIM1*, *HNRNPDL*
		miR-5192	*CBL*, *SOD2*, *POU2F1*, *UBE2V1*, *STX3*
		miR-4476	*PSMD11*, *SOD2*, *CFL1*, *IFNLR1*
		miR-1909-3p	*CANX*, *IRAK4*, *MCL1*
		miR-6840-3p	*CRK*, *IL6R*, *STAT2*
		miR-135b-5p	*STAT6*
		miR-6811-3p	*CCR2*
Cytokine signalling in immune system	1.23 × 10^6^	miR-16-5p	*BIRC5*, *MAP3K7*, *SOCS2*, *VEGFA*, *RPS6KA3*, *UBE2V1*, *BCL2*, *IRAK3*, *PIM1*, *HNRNPDL*, *NUP155*, *KRAS*, *CD44*
		miR-5192	*CBL*, *SOD2*, *POU2F1*, *UBE2V1*, *STX3*, *EDAR*
		miR-4476	*PSMD11*, *SOD2*, *CFL1*, *IFNLR1*
		miR-1909-3p	*CANX*, *IRAK4*, *MCL1*
		miR-6840-3p	*CRK*, *IL6R*, *STAT2*
		miR-6811-3p	*CCR2*, *PDE12*
		miR-135b-5p	*STAT6*

^1^ “Top five” over-represented pathways in the Reactome Pathway Database were defined as those with the lowest adjusted *p* values from the list of pathways with a BH-corrected *p* value ≤ 0.01 and with ≥3 RRMS-specific miRNAs having predictive mRNA targets inherent to the biological pathway.

**Table 2:** Demographic and clinical characteristics of study subjects.

Parameters	HealthCtl (*n* = 18)	HospCtl (*n* = 22)	CIS (*n* = 18)	RRMS (*n* = 16)	VI (*n* = 11)
Age: min–max (mean)	23–58 (34.0)	17–74 (43.1)	20–64 (31.9)	15–64 (35.6)	19–86 (46.5)
Gender: % Female	50	60	80	70	50
^1^ DMT (Yes: No)	N/A	N/A	1*:17	1**:15	N/A
^2^ EDSS: min–max (mean)	N/A	N/A	0.0–3.5 (1.7)	1.0–7.5 (2.3)	N/A
^3^ Stage (relapse: remission)	N/A	N/A	1:16 (N/V = 1)	16:0	N/A
^4^ Duration: min–max (mean)	N/A	N/A	N/A	1–156 (34)	N/V

^1^ DMT: disease-modifying therapy at the time of sample collection; ^2^ EDSS: Expanded Disability Status Scale score; ^3^ Disease stage at the time of sample collection; ^4^ Disease duration in months; N/A: not applicable; N/V: not available. * Fumarate. ** Natalizumab.

## Data Availability

The data presented in this study are available on request from the corresponding author.

## Supplementary Materials

The following are available at https://www.mdpi.com/article/10.3390/ijms23031664/s1.

## Abbreviations

BBB = blood–brain barrier; CIS = clinically isolated syndrome; CSF = cerebrospinal fluid; CNS = central nervous system; EVs = extracellular vesicles; HealthCtl = healthy controls; HospCtl = hospitalized controls; MS = multiple sclerosis; RRMS = relapsing–remitting multiple sclerosis; VI = viral inflammation.
