# Supplementary material for: Differential Expression of Serum Extracellular Vesicle miRNAs in Multiple Sclerosis: Disease-Stage Specificity and Relevance to Pathophysiology
Source: Int J Mol Sci. 2022 Jan 31;23(3):1664. doi: 10.3390/ijms23031664 (PMC8836256; doi:10.3390/ijms23031664)
Supplement: Supplementary file 1 [file ijms-23-01664-s001.zip › ijms-1564776-supplementary-done.pdf]

International Journal of
Molecular Sciences

MDPI

Supplementary Material

Methods

The validation of serum and CSF EV-rich fractions as collected by size exclusion chromatography was conducted using the following methods.

Tunable resistive pulse sensing: Pilot serum and CSF samples from healthy and hospitalized human subjects, respectively, were analyzed on a tunable resistive pulse sensing qNANO platform, following a standardized methodology [60] (Fig. S1A, B). Briefly, an NP100 membrane (Izon Science) was used, and the set-up calibrated using carboxylated particles (diameter = 110 nm). Size and concentration calibrations were performed using CPN100 standards at a concentration of 10^{10} particles/ml. The EV concentration at each size category was determined using a two-points pressure method, with pressures ranging between 0.3–2 kPa. All samples were run in triplicates. Measurements were carried out by Nanotechnology.life laboratories, Loughborough University (Loughborough, UK).

Transmission electron microscopy (TEM): Isolated EVs (5 µl) were applied onto a freshly glow discharged carbon film grid for 2 min, then washed with distilled de-ionized water and air dried. Negative staining was achieved by a double incubation with 5 µl of 1% uranyl acetate. Samples were viewed on a JEOL JEM-1400 TEM with an accelerating voltage of 120 kV. Images were collected using an EMSIS Xarosa digital camera running Radius software (Fig. S1C, D). The procedure was carried out at the Core Biotechnology Services Electron Microscopy Facility, University of Leicester (Leicester, UK).

Western blotting: Isolated EVs were lysed in radioimmunoprecipitation assay (RIPA) buffer containing protease inhibitor (cOmplete Mini, Merck). A Pierce BCA Protein Assay (Thermo Fischer) was used to assess protein concentration of individual (Fig. S1E, F) and pooled SEC fractions. Lysates were resolved onto a 12% polyacrylamide precast gel (Criterion TGX, Bio-Rad) using 1x sodium dodecyl sulfate (SDS) running buffer. Samples were then transferred onto a polyvinylidene difluoride (PVDF) membrane for 7 min at 25 V using the Trans-Blot Turbo Transfer system (Bio-Rad). Membranes were rinsed with PBS 1% Tween (PBS-T) and blocked with PBS-T containing 5% non-fat milk powder for 1 h at room temperature before overnight incubation with primary antibody at 4°C. Primary antibodies against the following EV marker proteins were used and at the dilutions indicated: Alix (1:5000, ab186429, Abcam), CD9 (1:2000, ab92726, Abcam), Flotilin-1 (1:1000, 610820, BD Transduction Laboratories), and CD-81 (1:250, MAB4615, R&D Systems). Membranes were then washed in PBS-T (3x 20 min), incubated for 1 h at room temperature with the appropriate horseradish peroxidase-conjugated secondary antibody (1:1000), and finally washed in PBS-T (3x 20 min). Lastly, membranes were incubated with detection solvent (Clarity ECL Western Detection System, Bio-Rad) and images were acquired using Chemidoc MP Imaging system (Bio-Rad) (Fig. S1G, H).

LDL-cholesterol assay: The extent of presence of contaminating low-density lipoproteins (LDL) in serum EV preparations was assessed by running 2.5 µl EV-rich fractions in the LDL-cholesterol (LDL-C) Assay Kit (Elabscience), according to the manufacturer´s instructions (Fig. S1I).

1. 60. Vogel, R.; Coumans, F.A.W.; Maltesen, R.G.; Böing, A.N.; Bonnington, K.E.; Broekman, M.L.; Broom, M.F.; Buzás, E.I.; Christiansen, G.; Hajji, N.; et al. A standardized method to determine the concentration of extracellular vesicles using tunable resistive pulse sensing. *J. Extracell. Vesicles* **2016**, *5*, 31242. https://doi.org/10.3402/jev.v5.31242

Results

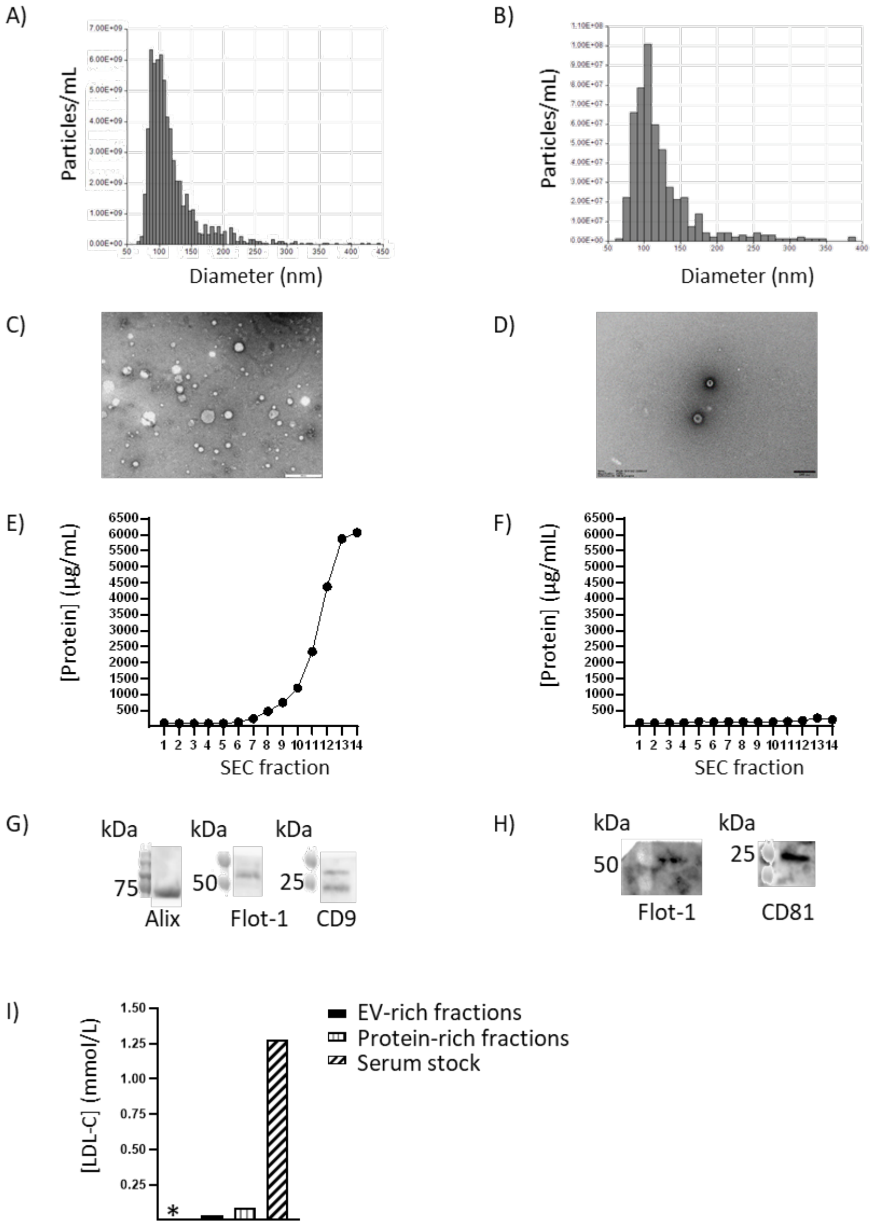

Figure S1. Validation of SEC-isolated serum and CSF EVs. (A, B) TRPS profile of a representative (A) serum EV-rich fraction (concentration: 6.37e10 particles/mL, mean size: 120 nm) and (B) CSF EV-rich fraction (concentration: 1.43e9 particles/mL, mean size: 127 nm). (C, D) Representative TEM image of (C) serum EVs and (D) CSF EVs; scale bar 200 nm. (E, F) Total protein quantification of individual SEC fractions (1-14) of (E) serum EVs and (F) CSF EVs. (G, H) Western blotting of (G) serum EV-rich fractions for the EV-specific protein markers Alix (75 kDa), Flotilin-1 (47 kDa), and CD9 (22 kDa), 40 µg protein per marker, and of (H) CSF EV-rich fractions for the EV-specific protein markers Flotilin-1 (47 kDa) and CD81 (25 kDa), 6 µg protein per marker. (I) LDL-C concentration for early fractions (1-5), EV-rich fractions (6-10), protein-rich fractions (11-15), and serum stock; * LDL-C levels in the early fractions were below detection threshold.

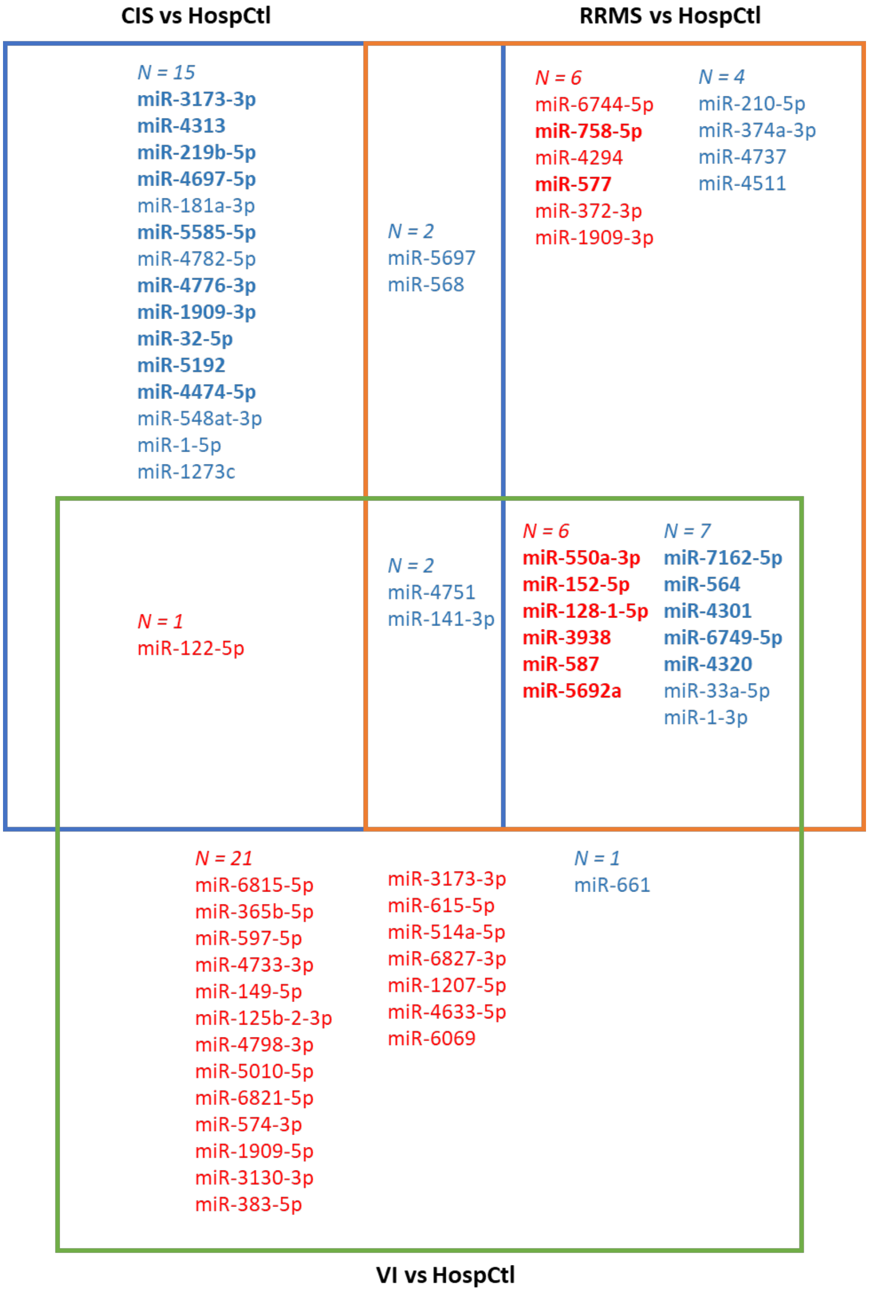

Figure S2. Venn diagram depicting the findings of pair-wise differential expression analysis (DEA) ($p < 0.01$, $\log_2$ fold change ≥ 1 or ≤ -1) of serum EV miRNomes in different subject groups. As a first step DEA was conducted for CIS vs HospCtl, RRMS vs HospCtl, and VI vs HospCtl. EV miRNAs indicated in blue were down-regulated in the corresponding disease state(s) relative to HospCtl, and miRNAs indicated in red were up-regulated in the corresponding disease state(s) relative to HospCtl. Next, DEA was conducted for CIS vs RRMS. EV miRNAs indicated in blue and bold were down-regulated in RRMS relative to HospCtl and CIS, and miRNAs indicated in red and bold were up-regulated in RRMS relative to HospCtl and CIS. Therefore, 15 down-regulated miRNAs were CIS-remission-specific relative to HospCtl and RRMS-relapse, and 12 miRNAs (6 up-regulated and 6 down-regulated) were RRMS-relapse-specific relative to HospCtl and CIS-remission.

Table S1. Comparison between miRNA-Seq and RT-qPCR expression data for two RRMS-specific miRNAs

	hsa-miR-16-5p		hsa-miR-451a	
	CIS	RRMS	CIS	RRMS
miRNA-Seq CPM mean	111182	219829	6627	14634
± SEM	14949	58466	1188	4415
Delta Cq mean	5.2	3.7	3.0	2.7
± SEM	0.20	0.24	0.19	0.21
Spearmann's r CPM against Delta Cq	-0.60	-0.78	-0.54	-0.83
Pearson's r p	0.04	0.0009	0.07	0.0003
miRNA-Seq F-test p	0.01		0.007	
miRNA-Seq CPM t-test p	0.1		0.1	
Delta Cq t-test p	< 0.0001		0.3	

CPM: normalized counts per million. *r*: Spearman rank-order correlation coefficient.

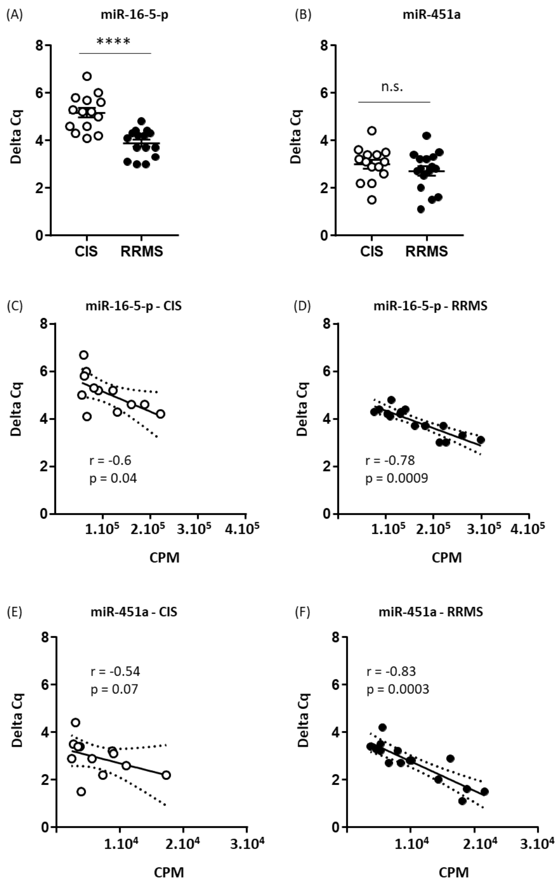

Figure S3. Validation of miRNA-Seq expression values using RT-qPCR. (A) miR-16-5p. (B) miR-451. ****p < 0.0001, two-tailed unpaired Student's *t*-test. n.s. = not significant. Individual values, mean and ± SEM values are shown. (C-F) Predictive association between miRNA-Seq normalized counts (CPM) and RT-PCR delta Cq values for miR-16-p within (C) CIS subjects and (D) RRMS subjects, and for miR-451a within (E) CIS subjects and (F) RRMS subjects. Scatter plots and the best-fit linear regression line (± 95% confidence intervals) are given.

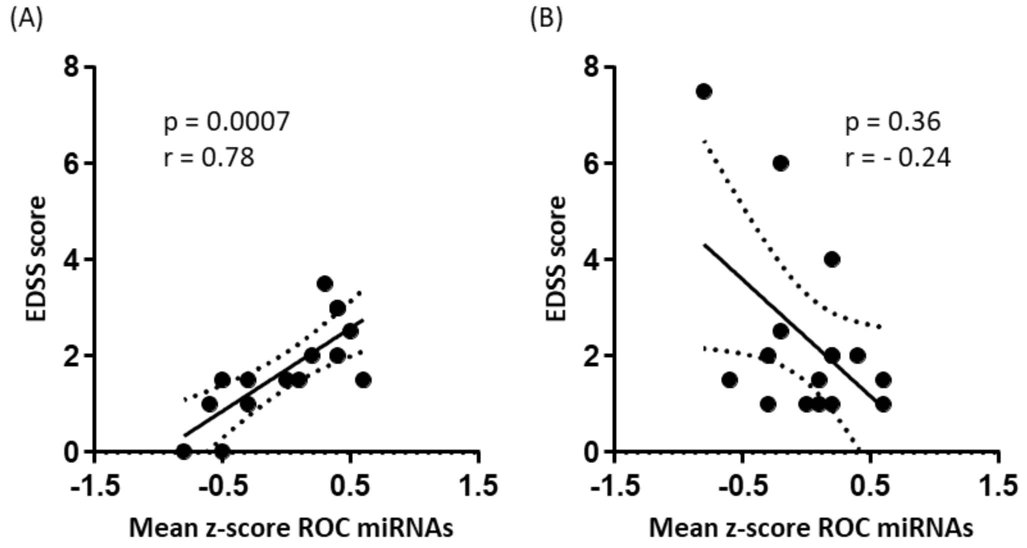

Figure S4. Predictive association between mean z-scores of the CIS-RRMS miRNA predictive panel and EDSS scores. Scatter plots and the best-fit linear regression line (± 95% confidence intervals) are given for (A) mean z-score of the CIS-RRMS miRNA predictive panel and EDSS scores of CIS patients and (B) mean z-score of the CIS-RRMS miRNA predictive panel and EDSS scores of RRMS patients.

Table S2. Complete list of over-represented pathways identified for validated target genes of the RRMS-specific miRNAs

Pathway	FDR	miRNAs	Target transcripts
Vesicle-mediated transport	2.14 × 10^8	hsa-miR-16-5p	*RAB9B,CAPZA2,DCTN5,GALNT1,RAB30,PPP6R3,RAB3IP,SBF1,TBC1D14,KIF2A,RACGAP1,KIF3B,KIF1B,TFRC,COL4A1*
		hsa-miR-6840-3p	*TRAPPC2,ACTR1A,FZD4,GNS,TBC1D24,GJC1,APOL1*
		hsa-miR-6811-3p	*RAB13,RAB30,PAFAH1B2,DENND6B*
		hsa-miR-5192	*CBL,CSNK1D,TBC1D13,SYT2,CALR*
		hsa-miR-4476	*RAB5B,ACTR2,PACSIN1*
		hsa-miR-135b-5p	*RAB3GAP2*
Membrane trafficking *(Vesicle-mediated transport)*	2.14 × 10^8	hsa-miR-6840-3p	*TRAPPC2,ACTR1A,FZD4,GNS,TBC1D24,GJC1*
		hsa-miR-6811-3p	*RAB13,RAB30,PAFAH1B2,DENND6B*
		hsa-miR-5192	*CBL,CSNK1D,TBC1D13,SYT2*
		hsa-miR-4476	*RAB5B,ACTR2,PACSIN1*
		hsa-miR-135b-5p	*RAB3GAP2*
Post-translational protein modification *(Metabolism of proteins)*	1.87 × 10^7	hsa-miR-16-5p	*RAB9B,CAPZA2,DCTN5,GALNT1,RAB30,PPP6R3,BIRC5,MAP3K7,SOCS2,NUP155,NCOR2,BRCA1,CUL3,FBXL20,B3GNT2,RAB23,GALNT7,ASXL1*
		hsa-miR-6840-3p	*TRAPPC2,ACTR1A,APOL1,MLEC,RCE1,SUMF2,GGCX,DCAF10,FOXK1,CPM*
		hsa-miR-4476	*RAB5B,PSMD11,PHC2,TFAP2A,USP12,IGFBP4*
		hsa-miR-1909-3p	*CANX,TFAP2B,SENP5,DPH2,WAC*
		hsa-miR-5192	*CSNK1D,CALR,ST6GAL2,RECK*

		hsa-miR-6811-3p	*RAB13,RAB30,KLHL21,PEX2*
		hsa-miR-135b-5p	*THBS2*
Signaling by Interleukins *(Immune system)*	1.87 × 10^7	hsa-miR-16-5p	*BIRC5,MAP3K7,SOCS2,VEGFA,RPS6KA3,UBE2V1,BCL2,IRAK3,PIM1,HNRNPDL*
		hsa-miR-5192	*CBL,SOD2,POU2F1,UBE2V1,STX3*
		hsa-miR-4476	*PSMD11,SOD2,CFL1,IFNLR1*
		hsa-miR-1909-3p	*CANX,IRAK4,MCL1*
		hsa-miR-6840-3p	*CRK,IL6R,STAT2*
		hsa-miR-135b-5p	*STAT6*
		hsa-miR-6811-3p	*CCR2*
Cytokine signalling in immune system *(Immune system)*	1.23 × 10^6	hsa-miR-16-5p	*BIRC5,MAP3K7,SOCS2,VEGFA,RPS6KA3,UBE2V1,BCL2,IRAK3,PIM1,HNRNPDL,NUP155,KRAS,CD44*
		hsa-miR-5192	*CBL,SOD2,POU2F1,UBE2V1,STX3,EDAR*
		hsa-miR-4476	*PSMD11,SOD2,CFL1,IFNLR1*
		hsa-miR-1909-3p	*CANX,IRAK4,MCL1*
		hsa-miR-6840-3p	*CRK,IL6R,STAT2*
		hsa-miR-6811-3p	*CCR2, PDE12*
		hsa-miR-135b-5p	*STAT6*
RNA Polymerase II transcritpion *(Transcription)*	7.78 × 10^6	hsa-miR-16-5p	*BIRC5,VEGFA,NCOR2,BRCA1,KRAS,ANAPC16,TBL1XR1,CCND2,CHEK1,ZC3H11A,CCNT2,TAF15,PIP4P1,PPM1D,ZNF691*
		hsa-miR-4476	*PSMD11,SOD2,PHC2,TFAP2A,TNRC6B,TXNIP,TFDP2,RNF111,CHTOP,GATAD2B,HDAC5,ZNF426*
		hsa-miR-6811-3p	*AGO3,ANAPC16,ZNF716,ZNF770,ZNF490,ZNF154,ZNF175*
		hsa-miR-6840-3p	*SKIL,OPRK1,INTS3,ZNF100,ZNF716,ZNF589*
		hsa-miR-5192	*SOD2,POU2F1,E2F6,CCND2,ZC3H11A,FANCC*
		hsa-miR-135b-5p	*TXNIP*
		hsa-miR-1909-3p	*TFAP2B*
Ca^{2+} pathway *(Signal transduction - Signaling by Wnt)*	1.15 × 10^5	hsa-miR-16-5p	*MAP3K7, KRAS*
		hsa-miR-4476	*TNRC6B*
		hsa-miR-6811-3p	*AGO3,PRKCA,PDE6A,PDE6B*
		hsa-miR-6840-3p	*FZD4, GNG4*
Innate immune system *(Immune system)*	1.65 × 10^5	hsa-miR-16-5p	*RAB9B,CAPZA2,MAP3K7,RPS6KA3,UBE2V1,BCL2,IRAK3,KRAS,CD44,GOLGA7,VCL,PYGB,PDXK*
		hsa-miR-6840-3p	*GNS,CRK,MLEC,RHOF,GM2A,RAP2B,POLR1D,SIKE1*
		hsa-miR-4476	*RAB5B,ACTR2,PSMD11,CFL1,TXNIP,ATOX1,FRK*
		hsa-miR-1909-3p	*IRAK4,ATOX1,VCL,NFASC*
		hsa-miR-6811-3p	*PAFAH1B2,CCR2,KLRD1,SVIP*
		hsa-miR-135b-5p	*STAT6, TXNIP*
		hsa-miR-5192	*UBE2V1, PKM*
Rab regulation of trafficking *(Vesicle-mediated transport - Clathrin-mediated endocytosis)*	4.46 × 10^5	hsa-miR-16-5p	*RAB9B,RAB3IP,SBF1,TBC1D14*
		hsa-miR-6811-3p	*RAB13, DENND6B*
		hsa-miR-6840-3p	*TRAPPC2, TBC1D24*
		hsa-miR-5192	*TBC1D13*
		hsa-miR-135b-5p	*RAB3GAP2*
		hsa-miR-4476	*RAB5B*

Beta-catening independent Wnt signaling pathway *(Signal transduction - Signaling by Wnt)*	0.0002	hsa-miR-6811-3p	*AGO3,PRKCA,PDE6A,PDE6B,PRICKLE1*
		hsa-miR-16-5p	*MAP3K7, KRAS*
		hsa-miR-4476	*PSMD11, TNRC6B*
		hsa-miR-6840-3p	*FZD4, GNG4*
Cellular repsonses to external stimuli *(Cellular response to stimuli)*	0.0002	hsa-miR-16-5p	*CAPZA2,DCTN5,VEGFA,RPS6KA3,NUP155,NCOR2,CUL3,ANAPC16,TBL1XR1,RPS24,RPL10,TXN2,HM13,HMGA2,NCOR2,CUL3,TBL1XR1,TXN2,HM13*
		hsa-miR-4476	*PSMD11,SOD2,PHC2,TNRC6B,TXNIP,TFDP2,ATOX1*
		hsa-miR-6811-3p	*AGO3,ANAPC16,BAG2*
		hsa-miR-135b-5p	*TXNIP*
		hsa-miR-1909-3p	*ATOX1*
		hsa-miR-5192	*SOD2*
		hsa-miR-6840-3p	*ACTR1A, RPL28*
Adaptive immune system *(Immune system)*	0.0004	hsa-miR-16-5p	*DCTN5,KIF2A,RACGAP1,KIF3B,MAP3K7,UBE2V1,CUL3,FBXL20,KRAS,SEC61A1,ANAPC13,TRIM4,UBE3C,CAPZA2*
		hsa-miR-6811-3p	*KLHL21,KLRD1,RAET1E*
		hsa-miR-6840-3p	*ACTR1A,TAP2,RNF14,CD300LG,NCR3LG1,BTN3A2*
		hsa-miR-4476	*PSMD11, RNF111*
		hsa-miR-5192	*CALR, UBE2V1*
		hsa-miR-1909-3p	*CANX*
Golgi to ER retrograde transport *(Vesicle-mediated transport - Clathrin-mediated endocytosis)*	0.0006	hsa-miR-16-5p	*CAPZA2,KIF2A,RACGAP1,KIF3B,KIF1B,GALNT1, DCTN5*
		hsa-miR-135b-5p	*RAB3GAP2*
		hsa-miR-6811-3p	*PAFAH1B2*
		hsa-miR-6840-3p	*ACTR1A*
Neutrophil degranulation *(Immune system - Innate)*	0.0007	hsa-miR-16-5p	*RAB9B,CD44,GOLGA7,VCL,PYGB,PDXK*
		hsa-miR-6840-3p	*GNS,MLEC,RHOF,GM2A,RAP2B*
		hsa-miR-4476	*RAB5B,ACTR2,PSMD11,FRK*
		hsa-miR-1909-3p	*VCL, NFASC*
		hsa-miR-6811-3p	*PAFAH1B2, SVIP*
		hsa-miR-5192	*PKM*
Intra-Golgi and retrograde Golgi to ER trafficking *(Vesicle-mediated transport - membrane trafficking)*	0.0007	hsa-miR-16-5p	*RAB9B,CAPZA2,DCTN5,GALNT1,RAB30,KIF2A,RACGAP1,KIF3B,KIF1B*
		hsa-miR-6811-3p	*RAB30, PAFAH1B2*
		hsa-miR-135b-5p	*RAB3GAP2*
		hsa-miR-6840-3p	*ACTR1A*
MAPK family signaling cascades *(Signal transduction)*	0.001	hsa-miR-16-5p	*CUL3,KRAS,GOLGA7,VCL,FNTA,ARL2*
		hsa-miR-6840-3p	*IL6R,RCE1,SPRED1*
		hsa-miR-4476	*PSMD11, TNRC6B*
		hsa-miR-5192	*FGFR1, CDC14B*

		hsa-miR-6811-3p	*AGO3, IGF2BP1*
		hsa-miR-1909-3p	*VCL*
Cell cycle	0.001	hsa-miR-16-5p	*PPP6R3,RAB3IP,SBF1,KIF2A,BIRC5,NUP155,BRCA1,ANAPC16,CCND2,CHEK1,TAOK1,ORC4,*
		hsa-miR-4476	*PSMD11,TFDP2,BUB3,TAOK1,SET*
		hsa-miR-5192	*CSNK1D,E2F6,CCND2*
		hsa-miR-6811-3p	*DENND6B,ANAPC16,PRKCA,SPC24,SPAST*
		hsa-miR-6840-3p	*ACTR1A,ARPP19,CTC1*
		hsa-miR-135b-5p	*RAB3GAP2*
		hsa-miR-4787-5p	*CENPO*
Rab GEFs exchange GTP for GDP on RABs *(Vesicle-mediated transport - membrane trafficking - Rab regulation of trafficking)*	0.001	hsa-miR-16-5p	*RAB9B,RAB3IP,SBF1*
		hsa-miR-6811-3p	*RAB13, DENND6B*
		hsa-miR-6840-3p	*TRAPPC2*
		hsa-miR-4476	*RAB5B*
		hsa-miR-135b-5p	*RAB3GAP2*
Signaling by Rho GTPases, Miro GTPases and RHOBTB3 *(Signal transduction)*	0.002	hsa-miR-16-5p	*CUL3,TAOK1,TXNL1,RAB9B,KIF2A,RACGAP1,TFRC,BIRC5*
		hsa-miR-4476	*BUB3,TAOK1,DBN1,ACTR2,CFL1*
		hsa-miR-6840-3p	*RHOF,ACBD5,ARHGAP11A*
		hsa-miR-6811-3p	*PRKCA,SPC24,EFHD2*
		hsa-miR-1909-3p	*SRGAP1, WDR81*
		hsa-miR-4787-5p	*CENPO*
		hsa-miR-5192	*RALBP1*
		hsa-miR-135b-5p	*NHS*
Metabolism of RNA	0.002	hsa-miR-16-5p	*NUP155,ZC3H11A,RPS24,RPL10,RCL1,UTP25,RBM28,EIF4B*
		hsa-miR-6840-3p	*RPL28,PRORP,RBM28,DCPS,CDKAL1*
		hsa-miR-4476	*PSMD11,CHTOP,SET,PRORP*
		hsa-miR-5192	*CSNK1D,ZC3H11A,HNRNPUL1*
		hsa-miR-6811-3p	*PRKCA,IGF2BP1,EXOSC2,FUS*
		hsa-miR-1909-3p	*HNRNPA3*
Signaling by TGFB family members *(Signal transduction)*	0.002	hsa-miR-16-5p	*NCOR2,CCNT2,ACVR2A*
		hsa-miR-4476	*TFDP2, RNF111*
		hsa-miR-5192	*CBL*
		hsa-miR-6840-3p	*SKIL*
		hsa-miR-135b-5p	*ACVR1B*
COPI-independent Gogli-to-ER retrograde traffic *(Vesicle-mediated transport - membrane trafficking)*	0.003	hsa-miR-16-5p	*CAPZA2,DCTN5,GALNT1*
		hsa-miR-6811-3p	*PAFAH1B2*
		hsa-miR-6840-3p	*ACTR1A*
		hsa-miR-135b-5p	*RAB3GAP2*

Cell cycle_Mitotic *(Cell cycle)*	0.003	hsa-miR-16-5p	*KIF2A,BIRC5,NUP155,ANAPC16,CCND2,TAOK1,ORC4*
		hsa-miR-4476	*PSMD11,TFDP2,BUB3,TAOK1,SET*
		hsa-miR-6811-3p	*ANAPC16,PRKCA,SPC24,SPAST*
		hsa-miR-5192	*CSNK1D,E2F6,CCND2*
		hsa-miR-4787-5p	*CENPO*
		hsa-miR-6840-3p	*ACTR1A, ARPP19*
Signaling by Wnt *(Signal transduction)*	0.003	hsa-miR-6811-3p	*AGO3,PRKCA,PDE6A,PDE6B,PRICKLE1*
		hsa-miR-16-5p	*MAP3K7,CUL3,KRAS*
		hsa-miR-4476	*PSMD11,TNRC6B,KREMEN1*
		hsa-miR-6840-3p	*FZD4,GNG4,PYGO1*
IL-4 and IL-13 signaling *(Immune system - cytokine signaling in immune system)*	0.003	hsa-miR-16-5p	*GALNT1,VEGFA,NUP155,NCOR2,TBL1XR1,CCNT2,TAF15,RPS24,RPL10*
		hsa-miR-135b-5p	*STAT6*
		hsa-miR-1909-3p	*MCL1*
		hsa-miR-5192	*POU2F1*
		hsa-miR-6840-3p	*IL6R*
Intracellular signaling by second messengers *(Signal transduction)*	0.005	hsa-miR-4476	*PSMD11,PHC2,TNRC6B,GATAD2B,HDAC5,FRK*
		hsa-miR-6811-3p	*AGO3, PRKCA*
		hsa-miR-6840-3p	*MTA3*
		hsa-miR-135b-5p	*PIP5K1A*
		hsa-miR-16-5p	*PHLPP2*
		hsa-miR-1909-3p	*IRAK4*
		hsa-miR-5192	*FGFR1*
Cellular response to chemical stress *(Cellular response to stress)*	0.008	hsa-miR-16-5p	*NCOR2, CUL3, TBL1XR1, TXN2, HM13*
		hsa-miR-4476	*PSMD11,SOD2,TXNIP,ATOX1*
		hsa-miR-135b-5p	*TXNIP*
		hsa-miR-5192	*SOD2*
		hsa-miR-1909-3p	*ATOX1*
M Phase *(Cell cycle)*	0.009	hsa-miR-16-5p	*KIF2A,BIRC5,NUP155,ANAPC16,TAOK1*
		hsa-miR-4476	*PSMD11,BUB3,TAOK1,SET*
		hsa-miR-6811-3p	*ANAPC16,PRKCA,SPC24,SPAST*
		hsa-miR-6840-3p	*ACTR1A, ARPP19*
		hsa-miR-4787-5p	*CENPO*
		hsa-miR-5192	*CSNK1D*
Transcriptional activity of SMAD2/SMAD3:SMAD4 heterodimer *(Transcription)*	0.009	hsa-miR-16-5p	*NCOR2, CCNT2*
		hsa-miR-4476	*TFDP2, RNF111*
		hsa-miR-6840-3p	*SKIL*
MHC I-mediated antigen processing and presentation *(Immune*	0.01	hsa-miR-16-5p	*UBE2V1,CUL3,FBXL20,SEC61A1,ANAPC13,TRIM4,UBE3C*
		hsa-miR-4476	*PSMD11, RNF111*
		hsa-miR-5192	*CALR, UBE2V1*
		hsa-miR-6840-3p	*TAP2, RNF14*
		hsa-miR-6811-3p	*KLHL21*

system - Adaptive)		hsa-miR-1909-3p	*CANX*
Hemostasis	0.01	hsa-miR-6840-3p	*CRK,GNG4,IGF1,PDE11A*
		hsa-miR-16-5p	*BIRC5,VEGFA,BCL2,PIM1*
		hsa-miR-6811-3p	*PRKCA, IGF1, TBXA2R*
		hsa-miR-4476	*CFL1,SDC4,SLC7A5*
		hsa-miR-1909-3p	*VCL*
		hsa-miR-5192	*FAM3C*
NR1H2 and NR1H3-mediated signaling *(Signal transduction - Signaling by nuclear receptors)*	0.01	hsa-miR-16-5p	*NCOR2, TBL1XR1*
		hsa-miR-4476	*TNRC6B*
		hsa-miR-6811-3p	*AGO3, SCD*
Signaling by TGF-beta receptor complex *(Signal transduction - Signaling by TGF-beta family members)*	0.01	hsa-miR-16-5p	*NCOR2, CCNT2*
		hsa-miR-4476	*TFDP2, RNF111*
		hsa-miR-5192	*CBL*
		hsa-miR-6840-3p	*SKIL*
Organelle biogenesis and mainainance	0.01	hsa-miR-6840-3p	*ACTR1A,SSTR3,CEP89,GABPB1,ATP5F1A*
		hsa-miR-16-5p	*RAB3IP,KIF3B,TBL1XR1,ARL3*
		hsa-miR-5192	*CSNK1D, SOD2*
		hsa-miR-6811-3p	*GABPB1*
		hsa-miR-4476	*UNC119B*
PTEN regulation *(Signal transduction - intracellular signaling by second messenger - PIP3 activates AKT signaling)*	0.01	hsa-miR-4476	*PSMD11,PHC2,TNRC6B,GATAD2B,HDAC5,FRK*
		hsa-miR-6811-3p	*AGO3*
		hsa-miR-6840-3p	*MTA3*
Infectious disease *(Disease)*	0.01	hsa-miR-6840-3p	*CRK,IL6R,STAT2,GNG4,RPL28,MTA3,VAMP1*
		hsa-miR-16-5p	*GALNT1, VEGFA, NUP155, NCOR2, TBL1XR1, CCNT2, TAF15, RPS24, RPL10*
		hsa-miR-4476	*ACTR2,PSMD11,TXNIP,GATAD2B*
		hsa-miR-5192	*CBL,SYT2,CALR*
		hsa-miR-135b-5p	*TXNIP*
		hsa-miR-1909-3p	*CANX*

Rab regulation of trafficking *(Vesicle-mediated transport - Clathrin-mediated endocytosis)*	4.46E-05	miR-16-5p	*RAB9B,RAB3IP,SBF1,TBC1D14*
		miR-6811-3p	*RAB13, DENND6B*
		miR-6840-3p	*TRAPPC2, TBC1D24*
		miR-5192	*TBC1D13*
		miR-135b-5p	*RAB3GAP2*
		miR-4476	*RAB5B*
Beta-catening independent Wnt signaling pathway *(Signal transduction - Signaling by Wnt)*	0.0002	miR-6811-3p	*AGO3,PRKCA,PDE6A,PDE6B,PRICKLE1*
		miR-16-5p	*MAP3K7, KRAS*
		miR-4476	*PSMD11, TNRC6B*
		miR-6840-3p	*FZD4, GNG4*
Cellular repsonses to external stimuli *(Cellular response to stimuli)*	0.0002	miR-16-5p	*CAPZA2,DCTN5,VEGFA,RPS6KA3,NUP155,NCOR2,CUL3,ANAPC16,TBL1XR1,RPS24,RPL10,TXN2,HM13,HMGA2,NCOR2,CUL3,TBL1XR1,TXN2,HM13*
		miR-4476	*PSMD11,SOD2,PHC2,TNRC6B,TXNIP,TFDP2,ATOX1*
		miR-6811-3p	*AGO3,ANAPC16,BAG2*
		miR-135b-5p	*TXNIP*
		miR-1909-3p	*ATOX1*
		miR-5192	*SOD2*
		miR-6840-3p	*ACTR1A, RPL28*
Adaptive immune system *(Immune system)*	0.0004	miR-16-5p	*DCTN5,KIF2A,RACGAP1,KIF3B,MAP3K7,UBE2V1,CUL3,FBXL20,KRAS,SEC61A1,ANAPC13,TRIM4,UBE3C,CAPZA2*
		miR-6811-3p	*KLHL21,KLRD1,RAET1E*
		miR-6840-3p	*ACTR1A,TAP2,RNF14,CD300LG,NCR3LG1,BTN3A2*
		miR-4476	*PSMD11, RNF111*
		miR-5192	*CALR, UBE2V1*
		miR-1909-3p	*CANX*
Golgi to ER retrograde transport *(Vesicle-mediated transport - Clathrin-mediated endocytosis)*	0.0006	miR-16-5p	*CAPZA2,KIF2A,RACGAP1,KIF3B,KIF1B,GALNT1, DCTN5*
		miR-135b-5p	*RAB3GAP2*
		miR-6811-3p	*PAFAH1B2*
		miR-6840-3p	*ACTR1A*
Neutrophil degranulation *(Immune system - Innate)*	0.0007	miR-16-5p	*RAB9B,CD44,GOLGA7,VCL,PYGB,PDXK*
		miR-6840-3p	*GNS,MLEC,RHOF,GM2A,RAP2B*
		miR-4476	*RAB5B,ACTR2,PSMD11,FRK*
		miR-1909-3p	*VCL, NFASC*
		miR-6811-3p	*PAFAH1B2, SVIP*
		miR-5192	*PKM*
Intra-Golgi and retrograde Golgi to ER trafficking *(Vesicle-mediated transport - membrane trafficking)*	0.0007	miR-16-5p	*RAB9B,CAPZA2,DCTN5,GALNT1,RAB30,KIF2A,RACGAP1,KIF3B,KIF1B*
		miR-6811-3p	*RAB30, PAFAH1B2*
		miR-135b-5p	*RAB3GAP2*
		miR-6840-3p	*ACTR1A*
MAPK family signaling cascades *(Signal transduction)*	0.001	miR-16-5p	*CUL3,KRAS,GOLGA7,VCL,FNTA,ARL2*
		miR-6840-3p	*IL6R,RCE1,SPRED1*
		miR-4476	*PSMD11, TNRC6B*
		miR-5192	*FGFR1, CDC14B*
		miR-6811-3p	*AGO3, IGF2BP1*
		miR-1909-3p	*VCL*
Cell cycle	0.001	miR-16-5p	*PPP6R3,RAB3IP,SBF1,KIF2A,BIRC5,NUP155,BRCA1,ANAPC16,CCND2,CHEK1,TAOK1,ORC4,*
		miR-4476	*PSMD11,TFDP2,BUB3,TAOK1,SET*
		miR-5192	*CSNK1D,E2F6,CCND2*
		miR-6811-3p	*DENND6B,ANAPC16,PRKCA,SPC24,SPAST*
		miR-6840-3p	*ACTR1A,ARPP19,CTC1*
		miR-135b-5p	*RAB3GAP2*
		miR-4787-5p	*CENPO*
Rab GEFs exchange GTP for GDP on RABs *(Vesicle-mediated transport - membrane trafficking - Rab regulation of trafficking)*	0.001	miR-16-5p	*RAB9B,RAB3IP,SBF1*
		miR-6811-3p	*RAB13, DENND6B*
		miR-6840-3p	*TRAPPC2*
		miR-4476	*RAB5B*
		miR-135b-5p	*RAB3GAP2*
Signaling by Rho GTPases, Miro GTPases and RHOBTB3 *(Signal transduction)*	0.002	miR-16-5p	*CUL3,TAOK1,TXNL1,RAB9B,KIF2A,RACGAP1,TFRC,BIRC5*
		miR-4476	*BUB3,TAOK1,DBN1,ACTR2,CFL1*
		miR-6840-3p	*RHOF,ACBD5,ARHGAP11A*
		miR-6811-3p	*PRKCA,SPC24,EFHD2*
		miR-1909-3p	*SRGAP1, WDR81*
		miR-4787-5p	*CENPO*
		miR-5192	*RALBP1*
		miR-135b-5p	*NHS*

Metabolism of RNA	0.002	miR-16-5p	*NUP155,ZC3H11A,RPS24,RPL10,RCL1,UTP25,RBM28,EIF4B*
		miR-6840-3p	*RPL28,PRORP,RBM28,DCPS,CDKAL1*
		miR-4476	*PSMD11,CHTOP,SET,PRORP*
		miR-5192	*CSNK1D,ZC3H11A,HNRNPUL1*
		miR-6811-3p	*PRKCA,IGF2BP1,EXOSC2,FUS*
		miR-1909-3p	*HNRNPA3*
Signaling by TGFB family members *(Signal transduction)*	0.002	miR-16-5p	*NCOR2,CCNT2,ACVR2A*
		miR-4476	*TFDP2, RNF111*
		miR-5192	*CBL*
		miR-6840-3p	*SKIL*
		miR-135b-5p	*ACVR1B*
COPI-independent Gogli-to-ER retrograde traffic *(Vesicle-mediated transport - membrane trafficking)*	0.003	miR-16-5p	*CAPZA2,DCTN5,GALNT1*
		miR-6811-3p	*PAFAH1B2*
		miR-6840-3p	*ACTR1A*
		miR-135b-5p	*RAB3GAP2*
Cell cycle_Mitotic *(Cell cycle)*	0.003	miR-16-5p	*KIF2A,BIRC5,NUP155,ANAPC16,CCND2,TAOK1,ORC4*
		miR-4476	*PSMD11,TFDP2,BUB3,TAOK1,SET*
		miR-6811-3p	*ANAPC16,PRKCA,SPC24,SPAST*
		miR-5192	*CSNK1D,E2F6,CCND2*
		miR-4787-5p	*CENPO*
		miR-6840-3p	*ACTR1A, ARPP19*
Signaling by Wnt *(Signal transduction)*	0.003	miR-6811-3p	*AGO3,PRKCA,PDE6A,PDE6B,PRICKLE1*
		miR-16-5p	*MAP3K7,CUL3,KRAS*
		miR-4476	*PSMD11,TNRC6B,KREMEN1*
		miR-6840-3p	*FZD4,GNG4,PYGO1*
IL-4 and IL-13 signaling *(Immune system - cytokine signaling in immune system)*	0.003	miR-16-5p	*GALNT1,VEGFA,NUP155,NCOR2,TBL1XR1,CCNT2,TAF15,RPS24,RPL10*
		miR-135b-5p	*STAT6*
		miR-1909-3p	*MCL1*
		miR-5192	*POU2F1*
		miR-6840-3p	*IL6R*
Intracellular signaling by second messengers *(Signal transduction)*	0.005	miR-4476	*PSMD11,PHC2,TNRC6B,GATAD2B,HDAC5,FRK*
		miR-6811-3p	*AGO3, PRKCA*
		miR-6840-3p	*MTA3*
		miR-135b-5p	*PIP5K1A*
		miR-16-5p	*PHLPP2*
		miR-1909-3p	*IRAK4*
		miR-5192	*FGFR1*
Cellular response to chemical stress *(Cellular response to stress)*	0.008	miR-16-5p	*NCOR2, CUL3, TBL1XR1, TXN2, HM13*
		miR-4476	*PSMD11,SOD2,TXNIP,ATOX1*
		miR-135b-5p	*TXNIP*
		miR-5192	*SOD2*
		miR-1909-3p	*ATOX1*
M Phase *(Cell cycle)*	0.009	miR-16-5p	*KIF2A,BIRC5,NUP155,ANAPC16,TAOK1*
		miR-4476	*PSMD11,BUB3,TAOK1,SET*
		miR-6811-3p	*ANAPC16,PRKCA,SPC24,SPAST*
		miR-6840-3p	*ACTR1A, ARPP19*
		miR-4787-5p	*CENPO*
		miR-5192	*CSNK1D*
Transcriptional activity of SMAD2/SMAD3:SMAD4 heterodimer *(Transcription)*	0.009	miR-16-5p	*NCOR2, CCNT2*
		miR-4476	*TFDP2, RNF111*
		miR-6840-3p	*SKIL*
MHC I-mediated antigen processing and presentation *(Immune system - Adaptive)*	0.01	miR-16-5p	*UBE2V1,CUL3,FBXL20,SEC61A1,ANAPC13,TRIM4,UBE3C*
		miR-4476	*PSMD11, RNF111*
		miR-5192	*CALR, UBE2V1*
		miR-6840-3p	*TAP2, RNF14*
		miR-6811-3p	*KLHL21*
		miR-1909-3p	*CANX*
Hemostasis	0.01	miR-6840-3p	*CRK,GNG4,IGF1,PDE11A*
		miR-16-5p	*BIRC5,VEGFA,BCL2,PIM1*
		miR-6811-3p	*PRKCA, IGF1, TBXA2R*
		miR-4476	*CFL1,SDC4,SLC7A5*
		miR-1909-3p	*VCL*
		miR-5192	*FAM3C*

NR1H2 and NR1H3-mediated signaling *(Signal transduction - Signaling by nuclear receptors)*	0.01	miR-16-5p	*NCOR2, TBL1XR1*
		miR-4476	*TNRC6B*
		miR-6811-3p	*AGO3, SCD*
Signaling by TGF-beta receptor complex *(Signal transduction - Signaling by TGF-beta family members)*	0.01	miR-16-5p	*NCOR2, CCNT2*
		miR-4476	*TFDP2, RNF111*
		miR-5192	*CBL*
		miR-6840-3p	*SKIL*
Organelle biogenesis and mainainance	0.01	miR-6840-3p	*ACTR1A,SSTR3,CEP89,GABPB1,ATP5F1A*
		miR-16-5p	*RAB3IP,KIF3B,TBL1XR1,ARL3*
		miR-5192	*CSNK1D, SOD2*
		miR-6811-3p	*GABPB1*
		miR-4476	*UNC119B*
PTEN regulation *(Signal transduction - intracellular signaling by second messenger - PIP3 activates AKT*	0.01	miR-4476	*PSMD11,PHC2,TNRC6B,GATAD2B,HDAC5,FRK*
		miR-6811-3p	*AGO3*
		miR-6840-3p	*MTA3*
Infectious disease *(Disease)*	0.01	miR-6840-3p	*CRK,IL6R,STAT2,GNG4,RPL28,MTA3,VAMP1*
		miR-16-5p	*GALNT1, VEGFA, NUP155, NCOR2, TBL1XR1, CCNT2, TAF15, RPS24, RPL10*
		miR-4476	*ACTR2,PSMD11,TXNIP,GATAD2B*
		miR-5192	*CBL,SYT2,CALR*
		miR-135b-5p	*TXNIP*
		miR-1909-3p	*CANX*
